# ﻿New species of *Labiobaetis* Novikova & Kluge from Thailand (Ephemeroptera, Baetidae)

**DOI:** 10.3897/zookeys.1258.166681

**Published:** 2025-11-06

**Authors:** Thomas Kaltenbach, Jean-Luc Gattolliat, Boonsatien Boonsoong, Chanaporn Suttinun

**Affiliations:** 1 Naturéum, Muséum cantonal des Sciences Naturelles, Département de zoologie, Palais de Rumine, Place Riponne 6, CH-1005 Lausanne, Switzerland Naturéum, Muséum cantonal des Sciences Naturelles Lausanne Switzerland; 2 Department of Ecology and Evolution, University of Lausanne (UNIL), CH-1015 Lausanne, Switzerland University of Lausanne (UNIL) Lausanne Switzerland; 3 Department of Zoology, Faculty of Science, Kasetsart University, Bangkok, Thailand Kasetsart University Bangkok Thailand; 4 Biodiversity Center Kasetsart University (BDCKU), Bangkok, Thailand Biodiversity Center Kasetsart University (BDCKU) Bangkok Thailand; 5 Faculty of Veterinary Medicine, Chiang Mai University, Chiang Mai 50100, Thailand Chiang Mai University Chiang Mai Thailand

**Keywords:** COI, integrative taxonomy, mayflies, morphology, Southeast Asia

## Abstract

Investigations of material collected by one of us (CS) between May 2017 and November 2023 from 70 localities in Thailand further increases our knowledge of the diversity of the genus *Labiobaetis* Novikova & Kluge, 1987 in Thailand and Southeast Asia in general. Eleven species have been identified using a combination of morphological and molecular analysis (COI). Nine are new to science, they are described and illustrated based on their larvae, and in one case, complemented by the male imago. Two of the new species belong to the *L. batakorum* species group, *L. mon***sp. nov.**, *L. lahu***sp. nov.**; three to the *L. numeratus* species group, *L. tenasserimensis***sp. nov.**, *L. angularis***sp. nov.**, *L. tonsator***sp. nov.**; one to the *L. operosus* species group, *L. nisaratae***sp. nov.**, and three to the *L. sumigarensis* species group, *L. karen***sp. nov.**, *L. septem***sp. nov.**, *L. ranongensis***sp. nov.** A key to all species of *Labiobaetis* from continental Southeast Asia is provided. Additionally, the genetic distances (COI; Kimura-2 parameter) including all species treated in this study are discussed. The total number of *Labiobaetis* species worldwide is augmented to approximately 170.

## ﻿Introduction

Southeast Asia is one of the regions with the highest biodiversity worldwide in general, and also specifically for mayflies. Considerable effort has been made in recent years to gain a better understanding of this yet understudied fauna, including studies focusing on the lesser known, but highly diverse mayfly family Baetidae. Particular emphasis was placed especially on Thailand, but also on the archipelagos of Indonesia and the Philippines, where new genera of Baetidae and many new species were discovered (e.g. Gattolliat 2012; Kaltenbach and Gattolliat 2019a, b, 2020, 2021; Kaltenbach et al. 2020b, 2021, 2022a, b, 2023a; Kluge 2020a, 2020b, 2022; studies on Thailand cited below). Thailand is located near the epicentre between China, the Indian subcontinent, and Southeast Asian islands. It is well-known for its geographical and ecological diversity, ranging from mountainous regions in the north to lowland floodplains in the centre, plateau areas shaped by tectonic processes in the northeastern and extensive coastal regions in the south. Thailand’s complex topography combined with its tropical climate gives rise to a wide variety of lotic habitats such as ri­vers and headwater streams, some of which are unique habitats (e.g. headwater streams on a limestone mountain range) (Ridd et al. 2011). Notably, they support an exceptionally diverse assemblage of mayflies especially in the family Baetidae. Over the last decade, two new genera and 17 new species were reported from Thailand (Kluge and Novikova 2017; Kluge et al. 2020; Kluge and Suttinun 2020; Sutthinun et al. 2018; Suttinun et al. 2020, 2021, 2022; Phlai-ngam et al. 2022a, b, 2024; Tungpairojwong et al. 2022; Bespalaya et al. 2023; Kaltenbach et al. 2023a). The high levels of endemism and species richness fostered by the complexity of these ecosystems, and influenced by both natural and man-made causes, make Thailand a key region for the study and conservation of freshwater biodiversity. Further collection efforts and studies are ongoing.

The genus *Labiobaetis* Novikova & Kluge, 1987 belongs to Baetidae, the most diverse family of Ephemeroptera. More than one fourth of all mayfly species worldwide (> 1200 species) are part of Baetidae (Sartori and Brittain 2015; Jacobus et al. 2019; updated by the authors). *Labiobaetis* is the richest genus of Baetidae, and one of the richest amongst mayflies in general, with ~160 previously described species (Lugo-Ortiz et al. 1999; Barber-James et al. 2013, Kaltenbach and Gattolliat 2018, 2019b, 2020, 2021 and citations therein; Kaltenbach et al. 2020a, 2022a and citations therein, 2023b; Sivaruban et al. 2022; Pandiaran et al. 2025). The distribution of *Labiobaetis* is nearly worldwide, except for the Neotropical realm, New Zealand, New Caledonia, and some remote islands. The history and concept of *Labiobaetis* were summarised in detail by Shi and Tong (2014) and Kaltenbach and Gattolliat (2018).

Recently, a comprehensive study on the Baetidae of Thailand was conducted by Suttinun (2021). Specimens were collected from 2017 to 2023 in 11 pro­vinces and 70 localities mainly in Western and Southern Thailand, and a few in Northern, Northeastern, and Eastern Thailand. Among many other genera of Baetidae, *Labiobaetis* was also treated in this study, without formerly describing and naming the new species. This contribution is based on the *Labiobaetis* material collected during this study (Suttinun 2021), and focuses on the formal taxonomic treatment, including the description of nine new species.

Given the extraordinary diversity in Thailand and despite the strong effort already done so far, it is reasonable to anticipate the discovery of numerous additional species through future studies in Thailand and in surrounding countries.

## ﻿Materials and methods

All specimens were preserved in 70%–96% ethanol. The dissection of larvae was done in Cellosolve (2-Ethoxyethanol) with subsequent mounting on slides with Euparal liquid, using an Olympus SZX7 stereomicroscope.

Photographs of larvae were taken using a Canon EOS 6D camera and processed with the programs Adobe Photoshop Lightroom (http://www.adobe.com) and Helicon Focus v. 5.3 (http://www.heliconsoft.com). Photographs of larval parts on slides were taken with an Olympus BX43 microscope equipped with an Olympus SC 50 camera and the program Olympus CellSense v. 4.1. All photographs were subsequently enhanced with Adobe Photoshop Elements 13.

The DNA of part of the specimens was extracted using non-destructive methods allowing subsequent morphological analysis (see Vuataz et al. 2011 for details). We amplified a 658 bp fragment of the mitochondrial gene cytochrome oxidase subunit 1 (COI) using the primers LCO 1490 and HCO 2198 (Folmer et al. 1994). Sequencing was done with Sanger’s method (Sanger et al. 1977). The genetic variability between specimens was estimated using Kimura-2-parameter distances (K2P; Kimura 1980), calculated with the program MEGA 11 (Tamura et al. 2021; http://www.megasoftware.net).

The GenBank accession numbers are given in Table 1; nomenclature of gene sequences follows Chakrabarty et al. (2013).

**Table 1.** Sequenced specimens.

Species	Specimen voucher	Specimen voucher	GPS coordinates	GenBank	GenSeq
	catalogue #	catalogue # (slide)		# (COI)	Nomenclature
*L. multus* (Sumatra)	GBIFCH00235847	–	00°34'25"S, 100°43'54"E	MN167323	genseq-4 COI
*L. multus*	GBIFCH00829311	GBIFCH01556161	07°34'32"N, 99°47'13"E	PX067793	genseq-4 COI
	GBIFCH00980855	–	07°29'33"N, 99°46'26"E	PX067795	genseq-4 COI
	GBIFCH00980853	–	07°43'44"N, 99°44'21"E	PX067794	genseq-4 COI
*L. mon* sp. nov.	GBIFCH00829315	GBIFCH01556160	12°03'50"N, 99°37'39"E	PX067748	genseq-2 COI
	GBIFCH00829307	GBIFCH00592518	13°30'33"N, 99°17'12"E	PX067749	genseq-2 COI
	GBIFCH00980852	–	13°30'57"N, 99°20'40"E	PX067746	genseq-2 COI
	GBIFCH00980854	–	12°58'42"N, 99°34'55"E	PX067745	genseq-2 COI
	GBIFCH00980857	–	12°58'42"N, 99°34'55"E	PX067747	genseq-1 COI
	GBIFCH00829296	–	12°52'36"N, 102°05'48"E	PX067744	genseq-2 COI
*L. lahu* sp. nov.	GBIFCH00829303	GBIFCH01223071	16°48'16"N, 99°01'19"E	PX067750	genseq-2 COI
	GBIFCH00829301	GBIFCH01223072	16°46'53"N, 99°01'16"E	PX067753	genseq-2 COI
	GBIFCH00829318	GBIFCH01223073	16°46'53"N, 99°01'16"E	PX067758	genseq-2 COI
	GBIFCH00829302	GBIFCH00596154	16°46'53"N, 99°01'16"E	PX067754	genseq-1 COI
	GBIFCH00829298	GBIFCH00607175	17°18'04"N, 101°46'33"E	PX067752	genseq-2 COI
	GBIFCH00829306	GBIFCH00607176	19°17'02"N, 98°58'05"E	PX067755	genseq-2 COI
	GBIFCH00829295	–	16°42'04"N, 98°30'41"E	PX067751	genseq-2 COI
	GBIFCH00980856	–	19°17'02"N, 98°58'05"E	PX067757	genseq-2 COI
	GBIFCH00980858	–	19°17'02"N, 98°58'05"E	PX067759	genseq-2 COI
	GBIFCH00980851	–	13°30'57"N, 99°20'40"E	PX067756	genseq-2 COI
*L. tenasserimensis* sp. nov.	GBIFCH00829286	–	13°31'27"N, 99°14'39"E	PX067774	genseq-2 COI
	GBIFCH00829275	–	13°31'27"N, 99°14'39"E	PX067772	genseq-2 COI
	GBIFCH00829285	–	09°41'27"N, 98°35'19"E	PX067773	genseq-2 COI
*L. angularis* sp. nov.	GBIFCH00829290	–	12°58'42"N, 99°34'55"E	PX067788	genseq-2 COI
	GBIFCH00829278	–	13°24'22"N, 99°16'44"E	PX067779	genseq-2 COI
	GBIFCH00829279	–	12°58'42"N, 99°34'55"E	PX067780	genseq-2 COI
	GBIFCH00829287	–	12°03'50"N, 99°37'39"E	PX067786	genseq-2 COI
	GBIFCH00829277	–	12°38'14"N, 99°30'59"E	PX067777	genseq-2 COI
	GBIFCH00829280	–	12°46'03"N, 99°34'54"E	PX067781	genseq-2 COI
	GBIFCH00829283	–	13°30'57"N, 99°20'40"E	PX067784	genseq-2 COI
	GBIFCH00829274	–	12°46'03"N, 99°34'54"E	PX067778	genseq-2 COI
	GBIFCH00829284	–	14°34'58"N, 98°34'52"E	PX067785	genseq-2 COI
	GBIFCH00829273	–	09°42'22"N, 98°34'39"E	PX067775	genseq-2 COI
	GBIFCH00829282	–	13°30'33"N, 99°17'12"E	PX067783	genseq-2 COI
	GBIFCH00829289	–	13°28'41"N, 99°14'55"E	PX067787	genseq-2 COI
	GBIFCH00829291	–	13°24'22"N, 99°16'44"E	PX067789	genseq-2 COI
	GBIFCH00829281	–	17°16'11"N, 101°35'51"E	PX067782	genseq-2 COI
	GBIFCH00829276	–	12°52'36"N, 102°05'48"E	PX067776	genseq-2 COI
*L. tonsator* sp. nov.	GBIFCH00829272	–	07°11'43"N, 100°04'18"E	PX067791	genseq-1 COI
L. cf. paraoperosus	GBIFCH00829310	GBIFCH01223074	10°03'54"N, 98°40'13"E	PX067797	genseq-4 COI
	GBIFCH00829312	GBIFCH00596156	10°03'54"N, 98°40'13"E	PX067796	genseq-4 COI
*L. nisaratae* sp. nov.	GBIFCH00829316	GBIFCH01223084	14°34'58"N, 98°34'52"E	PX067790	genseq-2 COI
*L. karen* sp. nov.	GBIFCH00829300	GBIFCH00592515	16°46'53"N, 99°01'16"E	PX067760	genseq-2 COI
	GBIFCH00980859	–	14°34'58"N, 98°34'52"E	PX067761	genseq-2 COI
*L. septem* sp. nov.	GBIFCH00829297	GBIFCH00596155	14°34'58"N, 98°34'52"E	PX067765	genseq-2 COI
	GBIFCH00829292	GBIFCH01221815	16°46'53"N, 99°01'16"E	PX067762	genseq-2 COI
	GBIFCH00829294	GBIFCH01221816	17°16'11"N, 101°35'51"E	PX067763	genseq-2 COI
	GBIFCH00975856	–	16°46'53"N, 99°01'16"E	PX067766	genseq-4 COI
	GBIFCH00975857	–	16°46'53"N, 99°01'16"E	PX067767	genseq-4 COI
	GBIFCH00975858	–	16°46'53"N, 99°01'16"E	PX067768	genseq-4 COI
	GBIFCH00980863	GBIFCH01221817	16°44'28"N, 99°02'43"E	PX067764	genseq-4 COI
L. cf. septem sp. nov.	GBIFCH00975853	–	19°19'19"N, 98°52'51"E	PX067798	genseq-4 COI
	GBIFCH00975854	–	19°19'19"N, 98°52'51"E	PX067799	genseq-4 COI
*L. ranongensis* sp. nov.	GBIFCH00980860	–	09°52'08"N, 98°37'32"E	PX067769	genseq-2 COI
	GBIFCH00980864	–	09°43'25"N, 98°36'29"E	PX067770	genseq-1 COI
	GBIFCH00975861	–	09°42'22"N, 98°34'39"E	PX067771	genseq-2 COI

The distribution maps were generated with the program SimpleMappr (https://simplemappr.net, Shorthouse 2010).

The dichotomous key was elaborated with the support of the program DKey v. 1.3.0 (http://drawwing.org/dkey, Tofilski 2018).

The terminology follows Kluge (2004) and Kluge (2005) (term “protopteron”).

### ﻿Abbreviations

**MZL**Naturéum, Muséum des Sciences Naturelles, Lausanne (Switzerland)

**VMCMU**Insect section of the Veterinary Anatomy and Pathology Museum Chiang Mai University, Chiang Mai (Thailand)

## ﻿Taxonomy

### ﻿List of *Labiobaetis* species from Thailand

*batakorum* group

1. *L. multus* (Müller-Liebenau, 1984) (new assignment to the group)
2. *Labiobaetis mon* sp. nov.
3. *Labiobaetis lahu* sp. nov.

*numeratus* group

1. *Labiobaetis tenasserimensis* sp. nov.
2. *Labiobaetis angularis* sp. nov.
3. *Labiobaetis tonsator* sp. nov.

*operosus* group

1. *Labiobaetis* cf. *paraoperosus* Kaltenbach & Gattolliat, 2018
2. *Labiobaetis nisaratae* sp. nov.

*sumigarensis* group

1. *Labiobaetis karen* sp. nov.
2. *Labiobaetis septem* sp. nov.
3. *Labiobaetis ranongensis* sp. nov.

### ﻿*Labiobaetis batakorum* species group (Kaltenbach and Gattolliat 2019b: 7)

**Diagnosis**. Dorsal surface of labrum with submarginal arc of simple setae, 1^st^ and 2^nd^ setae after submedian seta standing closely together; labial palp segment II with large, lobed distomedial protuberance; left mandible without setae at apex of mola; lingua of hypopharynx with well developed, short medial tuft of setae; distolateral process at scape well developed; paraproct distally not expanded; hind protoptera absent (*L. batakorum* species group s. str.) or present (*L. multus* and closely related species *L. mon* sp. nov. and *L. lahu* sp. nov.).

Probably, the *L. multus* species group should be included into the *L. tricolor* species group (Kluge et al. 2023). Here, we keep it separate until adults and eggs of at least one of the species from Southeast Asia are described.

The following species are known from continental Southeast Asia: *Labiobaetis multus* (Müller-Liebenau, 1984) (new assignment to the group), *Labiobaetis mon* sp. nov., *Labiobaetis lahu* sp. nov.

#### ﻿ Labiobaetis multus

Taxon classificationAnimaliaEphemeropteraBaetidae

(Müller-Liebenau, 1984)

F49FE553-2CCE-5AED-8C83-292B7D6F85D7

Fig. 1d

Baetis multus Müller-Liebenau, 1984: 263.
Labiobaetis multus (Müller-Liebenau): McCafferty and Waltz 1995: 21.

##### Material examined.

Thailand • 1 larva; Trang Prov., Na Yong Distr., Wang Nam Rab resort; 07°34'32"N, 99°47'13"E; 142 m; 18.v.2017; leg. C. Suttinun; on slide; GBIFCH01556161; VMCMU • 1 larva; Trang Prov., Yan Tha Khao Distr., Klong Tha Ped; 07°29'33"N, 99°46'26"E; 50 m; 18.v.2017; leg. C. Suttinun; on slide; GBIFCH00980855; VMCMU • 9 larvae; Trang Prov., Huai Yod Distr., Khao Lak waterfall; 07°43'44"N, 99°44'21"E; 66 m; 18.v.2017; leg. C. Suttinun; 9 in alcohol; GBIFCH00980853, GBIFCH00763798; MZL.

##### Diagnosis.

**Figure 1.**
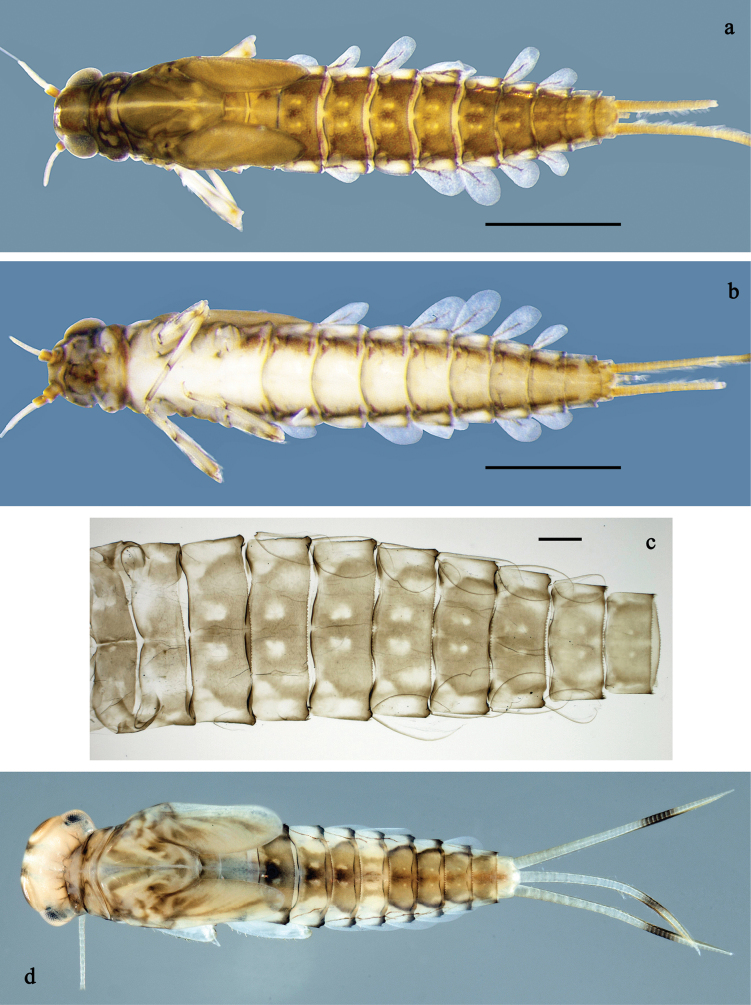
*Labiobaetis mon* sp. nov., larva: a. Habitus, dorsal view; b. Habitus, ventral view; c. Abdomen, dorsal view. *Labiobaetis multus* (Sumatra): d. Habitus, dorsal view. Scale bars: 1 mm (a, b); 100 µm (c).

**Larva.** Following combination of characters differentiate *L. multus* from other species of the group *batakorum*: head and thorax dorsally beige with grey-brown markings, abdomen dorsally grey-brown, laterally whitish with black markings, terga V and X brighter (Fig. 1d); hind protoptera well developed.

##### Comparison.

The species differs from *L. mon* sp. nov. and *L. lahu* sp. nov. mainly by the colour pattern of the larva (Fig. 1d). The figured larva is from Sumatra (Kaltenbach and Gattolliat 2019b), its conspecificity with material from Thailand is supported by morphology and a COI sequence. Additionally, Fig. 1d is in line with a picture of the type series of *L. multus* (Müller-Liebenau 1984: fig. 24).

##### Distribution.

Malaysia (Selangor), Indonesia (Sumatra), Thailand (Fig. 32a).

#### ﻿ Labiobaetis mon sp. nov.

Taxon classificationAnimaliaEphemeropteraBaetidae

F8930252-E356-591F-82D9-AC9241C71DE1

https://zoobank.org/80D6424A-DD90-455A-91D0-F8CCF3162AED

Figs 1a–c, 2, 3

##### Type material.

***Holotype*.** Thailand • larva; Phetchaburi Prov., Kaeng Krachan Distr., Huai Mae Kamoei; 12°58'42"N, 99°34'55"E; 119 m; 24.ii.2018; leg. C. Suttinun; on slide; GBIFCH00980857; VMCMU. ***Paratypes*.** 40 larvae; same data as holotype; 21 in alcohol; GBIFCH00980854, GBIFCH00763852; MZL; 19 in alcohol; GBIFCH00763797; VMCMU • 10 larvae; Ratchaburi Prov., Suan Phueng Distr., Swiss valley; 13°30'33"N, 99°17'12"E; 140 m; 26.v.2017; leg. C. Suttinun; 1 on slide; GBIFCH00592518; MZL; 9 in alcohol; GBIFCH00763808; VMCMU • 35 larvae; Ratchaburi Prov., Suan Phueng Distr., Pha Wo Tai; 13°30'57"N, 99°20'40"E; 118 m; 25.xi.2018; leg. C. Suttinun; 1 on slide; GBIFCH00980852; 34 in alcohol; GBIFCH00763799; MZL • 1 larva; Prachuab Khiri Khan Prov., Kui Buri Distr., Yang Chum; 12°04'40"N, 99°41'46"E; 57 m; 19.iv.2019; leg. C. Suttinun; on slide; GBIFCH00607177; VMCMU • 1 larva; Chanthaburi Prov., Khao Kitchakut Distr., Klong Krasue Yai; 12°52'36"N, 102°05'48"E; 38 m; 05.vi.2018; leg. C. Suttinun; on slide; GBIFCH00829296; MZL.

##### Other material.

Thailand • 1 larva; Prachuab Khiri Khan Prov., Kui Buri Distr., Huai Sam Rong; 100 m; 12°03'50"N, 99°37'39"E; 19.iv.2019; leg. C. Suttinun; on slide; GBIFCH01556160; VMCMU.

##### Diagnosis.

**Larva.** Following combination of characters differentiate *L. mon* sp. nov. from other species of the group *batakorum*: head and thorax dorsally mostly dark brown with indistinct brighter pattern; abdomen dorsally brown to dark brown, paler in middle area, segment V not much brighter; hind protoptera present, well developed.

##### Description.

**Figure 2.**
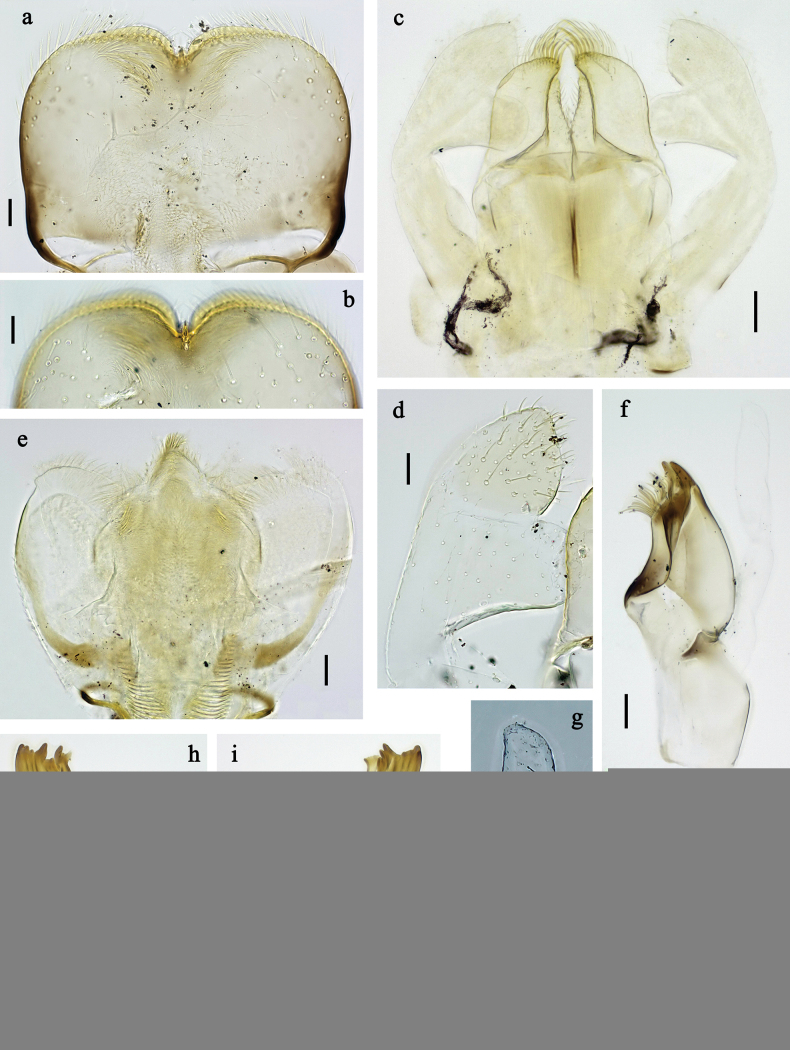
*Labiobaetis mon* sp. nov., larva morphology: a. Labrum; b. Section of labrum, dorsal focus; c. Labium; d. Labial palp; e. Hypopharynx and superlinguae; f. Maxilla; g. Maxillary palp; h. Left mandible i Right mandible. Scale bars: 20 µm (c, e, f, h, i); 10 µm (a, b, d, g).

**Figure 3.**
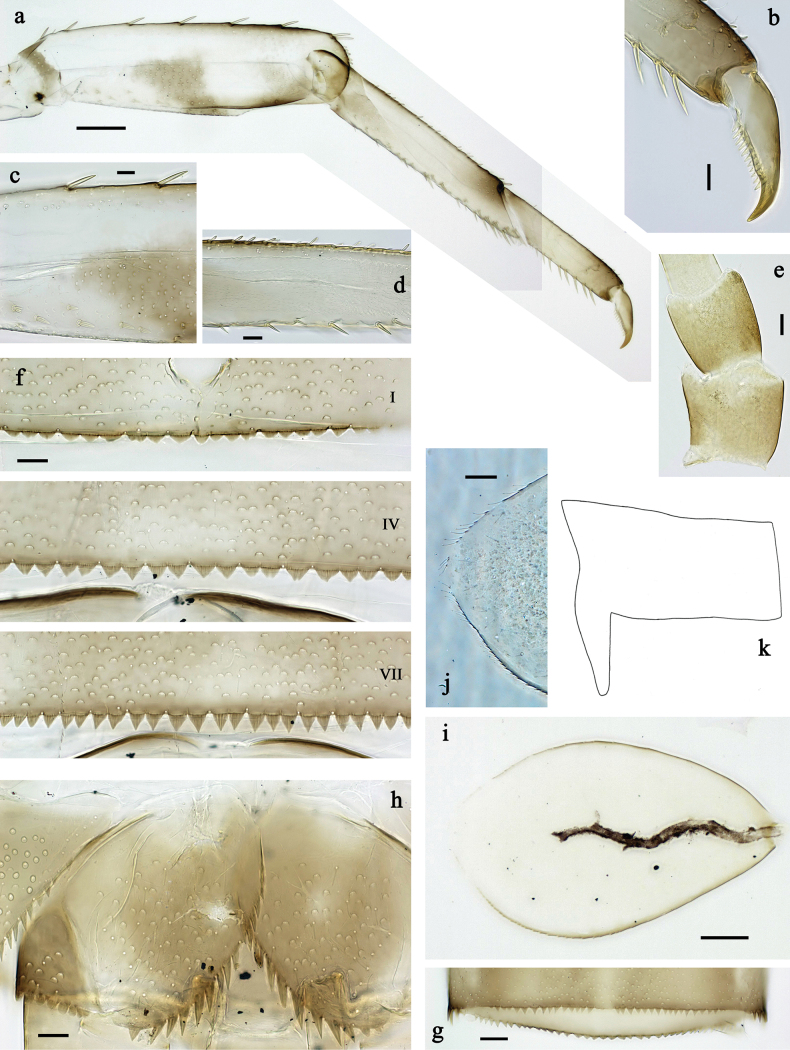
*Labiobaetis mon* sp. nov., larva morphology: a. Hind leg; b. Hind claw; c. Section of hind femur; d. Section of hind tibia; e. Antennal base; f. Posterior margins of abdominal terga; g. Abdominal segment IX, posterior margin of tergum and sternum; h. Paraprocts; i, j. Tergalius IV; k. Left half of metanotum with hind protopteron. Scale bars: 50 µm (a); 20 µm (i); 10 µm (b–h, j).

**Larva** (Figs 1a–c, 2, 3). Body length 3.6–4.8 mm. Cerci ~1/2 body length; paracercus ~1/3 of body length. Antenna: ~2× as long as head length.

***Colouration*** (Fig. 1a–c). Head and thorax head dorsally mostly dark brown with indistinct pattern as in Fig. 1a. Abdomen dorsally brown to dark brown, paler in middle area, segment X pale brown. Fore protoptera brown. Thorax and abdomen ventrally off-white to pale brown, laterally with dark brown marking. Legs off-white to pale brown, femur medially, distally, and basally with grey-brown markings, tibia, and tarsus distally darker. Caudalii yellow-brown, dark brown section in distomedial part.

***Antenna*** (Fig. 3e) with scape and pedicel sub cylindrical, distolateral process at scape well developed.

***Labrum*** (Fig. 2a, b). Sub-rectangular, length 0.7× maximum width. Distal margin with medial emargination and small process. Dorsally with medium, fine, simple setae scattered over surface; pair of submedian setae, and submarginal arc of six long, simple setae on each side, 1^st^ and 2^nd^ setae closely together. Ventrally with marginal row of setae composed of lateral and anterolateral long, feathered setae and medial long, bifid setae.

***Right mandible*** (Fig. 2i). Incisor and kinetodontium fused. Incisor with three denticles; kinetodontium with four denticles, inner margin of innermost denticle with row of thin setae. Prostheca robust, apically denticulate. Margin between prostheca and mola convex, smooth. Tuft of setae on proximal corner of mola present.

***Left mandible*** (Fig. 2h). Incisor and kinetodontium fused. Incisor with four denticles, kinetodontium with three denticles. Prostheca robust, apicolaterally with small denticles and comb-shaped structure. Margin between prostheca and mola slightly convex, smooth. Tuft of setae on proximal corner of mola present.

Both mandibles with lateral margins almost straight.

***Hypopharynx and superlinguae*** (Fig. 2e). Lingua approx. as long as superlinguae. Lingua longer than broad; medial tuft of stout setae well developed, short; medially expanded. Superlinguae with lateral margins rounded; fine, long, simple setae along distal margin.

***Maxilla*** (Fig. 2f, g). Galea-lacinia ventrally with two simple, apical setae below canines. Medially with one feathered, spine-like seta and four short to long, simple setae. Maxillary palp longer than length of galea-lacinia; 2-segmented; palp segment II approx. as long as segment I; setae on maxillary palp fine, simple, scattered over surface of segments I and II; apex of last segment with distolateral excavation, apically rounded.

***Labium*** (Fig. 2c, d). Glossa basally broad, narrowing toward apex; shorter than paraglossa; inner margin with ~9 spine-like seta; apex with two long and one medium, robust, apically pectinate setae; outer margin with ~4 spine-like setae; ventral surface with fine, simple, scattered setae. Paraglossa sub-rectangular, slightly curved inward; apex rounded; with three rows of long, robust, distally pectinate setae in apical area and ~2 short, simple setae in anteromedial area; dorsally with five or six long, spine-like setae near inner margin. Labial palp with segment I approx. as long as length of segments II and III combined. Segment II with broadly rounded, thumb-like, distomedial protuberance; distomedial protuberance 0.7× width of base of segment III; dorsally with row of ~4 spine-like setae near outer margin. Segment III conical; length approx. as maximal width; ventrally covered with short, spine-like, simple setae and short, fine, simple setae.

***Hind protoptera*** (Fig. 3k) present, well developed.

***Legs*** (Fig. 3a–d). Ratio of foreleg segments 1.3:1.0:0.7:0.2, middle leg 1.3:1.0:0.6:0.3, hind leg 1.4:1.0:0.7:0.2. ***Femur***. Fore femur length ~3× maximum width, middle and hind femur less wide. Outer margin with a row of 7–12 spine-like setae; length of setae ~0.2× maximum width of femur. Apex rounded, with pair of spine-like setae and short, stout, apically blunt setae. Stout, lanceolate, pointed setae scattered along inner margin; femoral patch reduced or rudimentary on foreleg and well developed on middle and hind leg. ***Tibia.*** Outer margin with row of short, stout, apically rounded setae, distalmost seta larger. Inner margin with row of medium spine-like setae, on apex tuft of fine, simple setae. Patella-tibial suture present on basal 2/3 area. ***Tarsus.*** Outer margin with row of short, stout, apically rounded setae. Inner margin with row of curved, spine-like setae increasing in length distally. ***Claw*** with one row of ~12 denticles; distally pointed.

***Abdominal terga*** (Fig. 3f, g). Surface with irregular rows of U-shaped scale bases and fine, simple, scattered setae. Posterior margin of terga: I–IX with triangular spines, wide and short on I, less wide and longer toward end of abdomen.

***Abdominal sterna*** (Fig. 3g). Posterior margin of sterna: I–VI smooth, without spines; VII–IX with small, triangular spines.

***Tergalii*** (Fig. 3i, j). Present on segments I–VII. Margin with small denticles intercalating fine, simple setae. Tracheae mainly limited to trunk. Tergalius I as long as length of 2/3 II; tergalius IV as long as segments V and 1/2 VI combined; tergalius VII slightly longer than segment VIII.

***Paraproct*** (Fig. 3h). Distally not expanded, with 9–15 stout, marginal spines. Surface scattered with U-shaped scale bases and fine, simple setae. Cercotractor with numerous small, marginal spines.

##### Imago.

Unknown.

##### Etymology.

The species is dedicated to the indigenous Mon people in Thailand.

##### Distribution.

Thailand (Fig. 32a).

#### ﻿ Labiobaetis lahu sp. nov.

Taxon classificationAnimaliaEphemeropteraBaetidae

7155FB80-A354-5232-A918-5C7B873E45BE

https://zoobank.org/16E1B9B6-10C2-4C03-909A-1C565F99276A

Figs 4, 5, 6

##### Type material.

***Holotype*.** Thailand • larva; Tak Prov., Mueang Distr., Klong Lan Sang; 16°46'53"N, 99°01'16"E; 253 m; 25.xii.2017; leg. C. Suttinun; on slide; GBIFCH00596154; VMCMU. ***Paratypes*.** 2 larvae; same data as holotype; on slides; GBIFCH01223072, GBIFCH01223073; MZL • 2 larvae; Tak Prov., Mueang Distr., Oum Yom; 06°48'16"N, 99°01'19"E; 249 m; 26.xii.2017; leg. C. Suttinun; 1 on slide; GBIFCH01223071; 1 in alcohol; GBIFCH00763789; VMCMU • 8 larvae; Loei Prov., Wang Saphung Distr., Loei River; 17°18'04"N, 101°46'33"E; 256 m; 18.xii.2018; leg. C. Suttinun; 1 on slide; GBIFCH00607175; 7 in alcohol; GBIFCH00763787, GBIFCH00763790; VMCMU • 22 larvae; Chiang Mai Prov., Chiang Dao Distr., Kaeng Pantao; 19°17'02"N, 98°58'05"E; 403 m; 21.xi.2018 and 05.xi.2023; leg. C. Suttinun; 1 on slide; GBIFCH00607176; MZL; 21 in alcohol; GBIFCH00980856, GBIFCH00980858, GBIFCH00763791, GBIFCH00763786, GBIFCH00763871, GBIFCH00763874; VMCMU • 63 larvae; Tak Prov., Mae Sot Distr., Moei River; 16°42'04"N, 98°30'41"E; 194 m; 27.xii.2017; leg. C. Suttinun; in alcohol; GBIFCH00829295, GBIFCH00763785, GBIFCH00763788; VMCMU; GBIFCH00763851, GBIFCH00763873; MZL • 23 larvae; Ratchaburi Prov., Suan Phueng Distr., Pha Wo Tai; 13°30'57"N, 99°20'40"E; 118 m; 25.xi.2018; leg. C. Suttinun; in alcohol; GBIFCH00980851, GBIFCH00763807; VMCMU.

##### Diagnosis.

**Figure 4.**
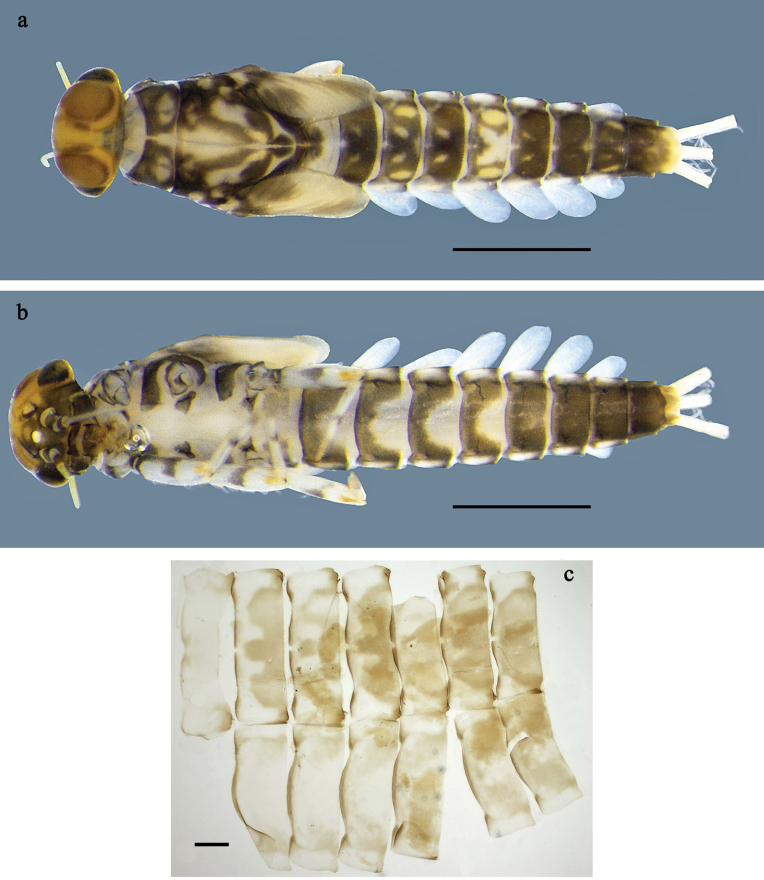
*Labiobaetis lahu* sp. nov., larva: a. Habitus, dorsal view; b. Habitus, ventral view; c. Abdomen, dorsal and ventral view. Scale bars: 1 mm (a, b); 100 µm (c).

**Larva.** Following combination of characters differentiate *L. lahu* sp. nov. from other species of the group *batakorum*: thorax and abdomen dorsally dark grey with yellowish pattern as in Fig. 4a, especially yellowish oval markings on terga III and V; legs with reddish-brown spot distally on femur; hind protoptera present, well developed.

##### Description.

**Figure 5.**
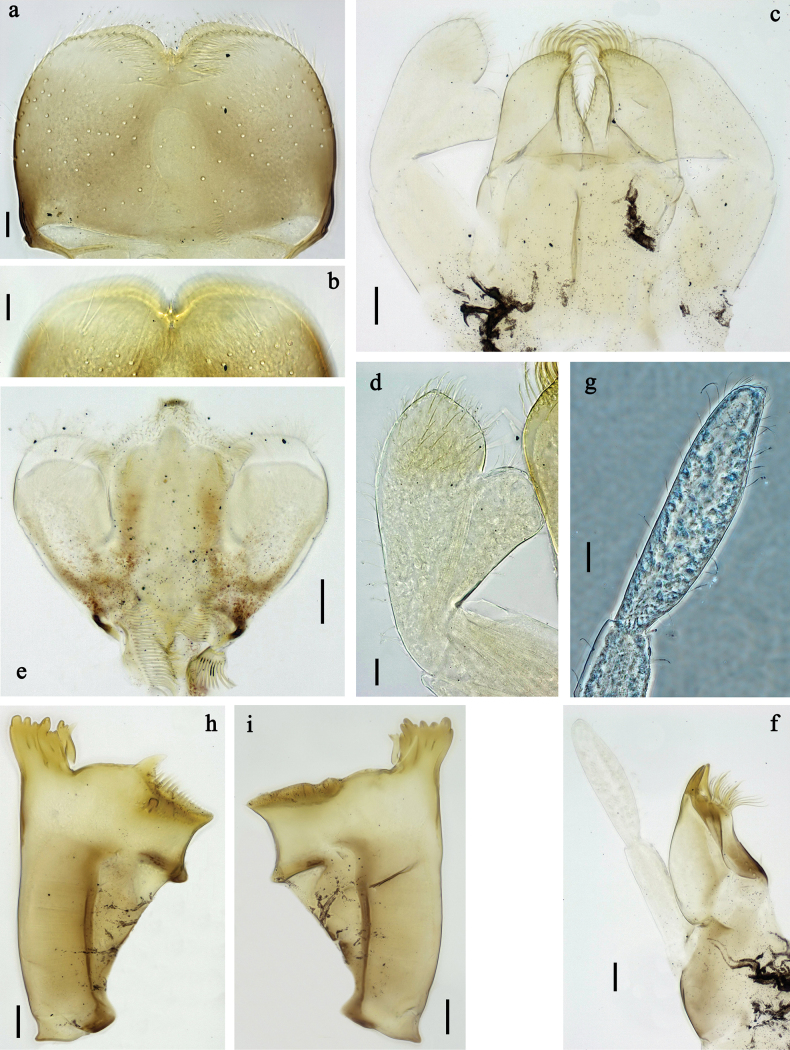
*Labiobaetis lahu* sp. nov., larva morphology: a. Labrum; b. Section of labrum, dorsal focus; c. Labium; d. Labial palp; e. Hypopharynx and superlinguae; f. Maxilla; g. Maxillary palp; h. Left mandible; i. Right mandible. Scale bars: 20 µm (c, e, f, h, i); 10 µm (a, b, d, g).

**Figure 6.**
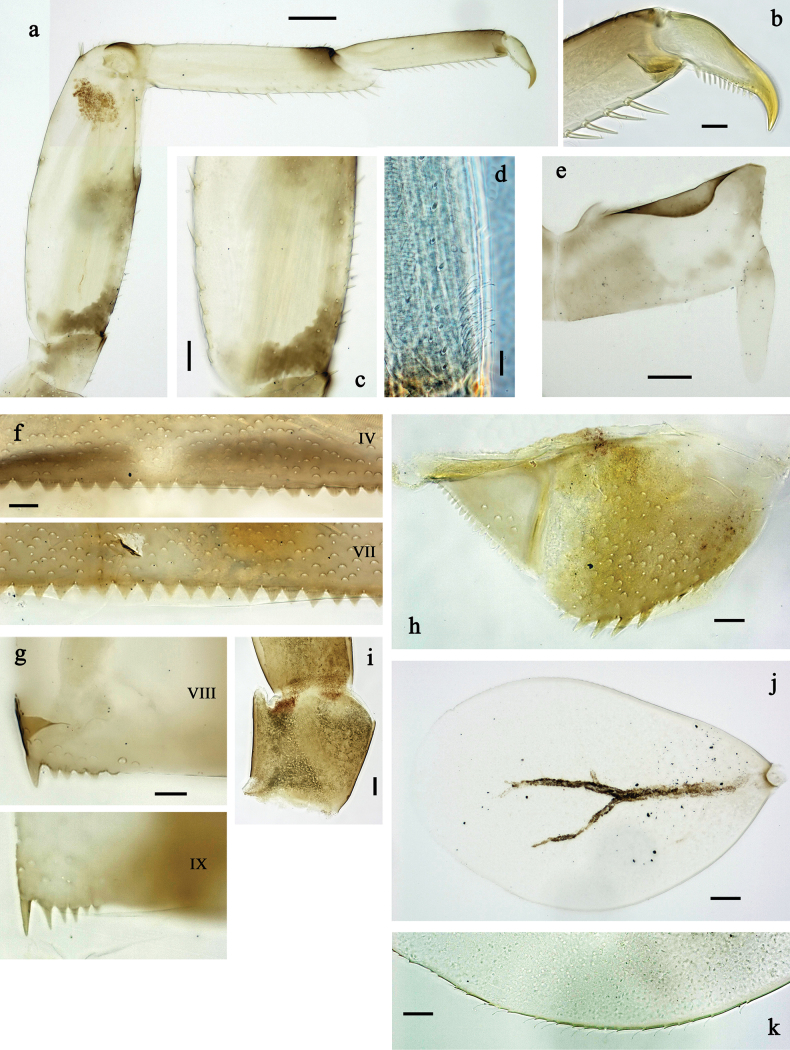
*Labiobaetis lahu* sp. nov., larva morphology: a. Hind leg; b. Fore claw; c. Section of hind femur; d. Section of fore femur with femoral patch; e. Right half of metanotum with hind protopteron; f. Posterior margins of abdominal terga; g. Abdominal terga VIII and IX, posterolateral margin; h. Paraproct; i. Antennal base; j, k. Tergalius V. Scale bars: 50 µm (a, e); 20 µm (j); 10 µm (b–d, f–i).

**Larva** (Figs 4–6). Body length 4.2–5.0 mm. Cerci ~0.4 body length, paracercus ~0.3× body length. Antenna: ~2× as long as head length.

***Colouration*** (Fig. 4a–c). Thorax and abdomen dorsally dark grey with distinct yellowish markings as in Fig. 1a; abdominal segments V and X brighter. Fore protoptera yellowish and grey. Thorax and abdomen ventrally whitish-yellow; abdominal segments II–V with dark grey, arched marking; segments VI–IX dark grey. Legs off-white to yellowish, femur medially and basally with grey markings, distally with distinct reddish-brown spot, tibia and tarsus distally grey. Caudalii whitish-yellow, dark brown section in distomedial part.

***Antenna*** (Fig. 6i) with scape and pedicel sub cylindrical, distolateral process at scape well developed.

***Labrum*** (Fig. 5a, b). Sub-rectangular, length 0.7× maximum width. Distal margin with medial emargination and small process. Dorsally with medium, fine, simple setae scattered over surface; pair of submedian setae, and submarginal arc of four or five long, simple setae on each side, 1^st^ and 2^nd^ setae closely together. Ventrally with marginal row of setae composed of lateral and anterolateral long, feathered setae and medial long, bifid setae.

***Right mandible*** (Fig. 5i). Incisor and kinetodontium fused. Incisor with four denticles; kinetodontium with four denticles, inner margin of innermost denticle with row of thin setae. Prostheca robust, apically denticulate. Margin between prostheca and mola slightly convex, smooth. Tuft of setae on proximal corner of mola present.

***Left mandible*** (Fig. 5h). Incisor and kinetodontium fused. Incisor with four denticles, kinetodontium with three denticles. Prostheca robust, apicolaterally with small denticles and comb-shaped structure. Margin between prostheca and mola slightly convex, smooth. Tuft of setae on proximal corner of mola absent.

Both mandibles with lateral margins almost straight.

***Hypopharynx*** and ***superlinguae*** (Fig. 5e). Lingua longer than superlinguae. Lingua longer than broad; medial tuft of stout setae well developed, short; in distal 1/2 expanded. Superlinguae with lateral margins rounded; fine, long, simple setae along distal margin.

***Maxilla*** (Fig. 5f, g). Galea-lacinia ventrally with two simple, apical setae below canines. Medially with one feathered, spine-like seta and four short to long, simple setae. Maxillary palp longer than length of galea-lacinia; 2-segmented; palp segment II approx. as long as segment I; setae on maxillary palp fine, simple, scattered over surface of segments I and II; apex of last segment with distolateral excavation, apically constricted.

***Labium*** (Fig. 5c, d). Glossa basally broad, narrowing toward apex; shorter than paraglossa; inner margin with ~9 spine-like seta; apex with three long, robust, apically pectinate setae; outer margin with ~4 spine-like setae; ventral surface with fine, simple, scattered setae. Paraglossa sub-rectangular, slightly curved inward; apex rounded; with three rows of long, robust, distally pectinate setae in apical area and one short, simple seta in anteromedial area; dorsally with five long, spine-like setae near inner margin. Labial palp with segment I ~0.8× length of segments II and III combined. Segment II with broadly rounded, thumb-like, distomedial protuberance; distomedial protuberance 0.7× width of base of segment III; dorsally with row of ~4 spine-like setae near outer margin. Segment III conical; length ~0.8× maximal width; ventrally covered with short, spine-like, simple setae and short, fine, simple setae.

***Hind protoptera*** (Fig. 6e) present, well developed.

***Legs*** (Fig. 6a–d). Ratio of foreleg segments 1.4:1.0:0.6:0.2, middle leg 1.3:1.0:0.6:0.2, hind leg 1.2:1.0:0.7:0.2. ***Femur***. Femur length ~3× maximum width. Outer margin with a row of 8–10 spine-like setae; length of setae ~0.2× maximum width of femur. Apex rounded, with pair of spine-like setae and short, stout, apically blunt setae. Stout, lanceolate, pointed setae scattered along inner margin; femoral patch well developed on fore and middle leg, reduced or rudimentary on hind leg. ***Tibia.*** Outer margin with row of short, stout, apically rounded setae, distalmost seta larger. Inner margin with row of medium spine-like setae; on apex tuft of fine, simple setae. Patella-tibial suture present on basal 1/2. ***Tarsus.*** Outer margin with row of short, stout, apically rounded setae. Inner margin with row of curved, spine-like setae increasing in length distally. ***Claw*** with one row of 12 or 13 denticles; distally pointed.

***Abdominal terga*** (Fig. 6f, g). Surface with irregular rows of U-shaped scale bases and fine, simple, scattered setae. Posterior margin of terga: I–IX with triangular spines, wide and short on I, less wide and longer toward end of abdomen; terga VIII and IX additionally with posterolateral spines.

***Abdominal sterna.*** Posterior margin of sterna: I–VI smooth, without spines; VII–IX with small, triangular spines.

***Tergalii*** (Fig. 6j, k). Present on segments I–VII. Margin with small denticles intercalating fine, simple setae. Tracheae limited to trunk and few branches. Tergalius I as long as length of 1/2 II; tergalius IV as long as segments V and 1/3 VI combined; tergalius VII approx. as long as segment VIII.

***Paraproct*** (Fig. 6h). Distally not expanded, with ~14 stout, marginal spines. Surface scattered with U-shaped scale bases and fine, simple setae. Cercotractor with numerous small, marginal spines.

##### Imago.

Unknown.

##### Etymology.

The species is dedicated to the indigenous Lahu people in Thailand.

##### Distribution.

Thailand (Fig. 32a).

### ﻿*Labiobaetis numeratus* species group (Kaltenbach and Gattolliat 2019b: 62)

**Diagnosis**. Dorsal surface of labrum with submarginal row of simple setae, 1^st^ and 2^nd^ setae after submedian seta closely together; labial palp segment II with thumb-like protuberance; right mandible with pronounced hump between prostheca and mola, with thin setae basally at mola; distal margin of left mandible usually convex; maxillary palp segment II longer than segment I, usually bent; antennal scape without distolateral process; superlinguae strongly sclerotised along basal margin; glossae with robust setae along inner margin; vestigial hind protoptera; tergalii on abdominal segments II–VII; outer margin of femur with partial 2^nd^ row of setae in proximal 1/2; tibia ventral margin with much longer seta distally at suture.

The following species are known from continental Southeast Asia: *Labiobaetis numeratus* (Müller-Liebenau, 1984), *Labiobaetis tenasserimensis* sp. nov., *Labiobaetis angularis* sp. nov., *Labiobaetis tonsator* sp. nov.

#### ﻿ Labiobaetis tenasserimensis sp. nov.

Taxon classificationAnimaliaEphemeropteraBaetidae

CAF84DFA-4F13-5611-A2FE-21B55E3AF39C

https://zoobank.org/4C747AF6-9599-417A-9523-C6D07647170B

Figs 7, 8, 9

##### Type material.

***Holotype*.** Thailand • larva; Ratchaburi Prov., Suan Phueng Distr., Bo Klueng; 13°31'27"N, 99°14'39"E; 180 m; 25.xi.2018; leg. C. Suttinun; on slide; GBIFCH00763877; VMCMU. ***Paratypes*.** 7 larvae; same data as holotype; 3 on slides; GBIFCH00829286; GBIFCH00829275; MZL; GBIFCH00607180; VMCMU; 4 in alcohol; GBIFCH00763853; MZL • 3 larvae; Ranong Prov., Mueang Distr., Rak Loi Waterfall; 09°41'27"N, 98°35'19"E; 78 m; 21.vi.2018; leg. C. Suttinun; 1 on slide; GBIFCH00829285; 2 in alcohol; GBIFCH00763855; VMCMU.

##### Diagnosis.

**Larva.** Following combination of characters differentiate *L. tenasserimensis* sp. nov. from other species of the group *numeratus*: abdomen dorsally rather uniform brown; hypopharynx with well-developed medial tuft; labial palp segment II lobed, segment III rather short; spines at posterior margin of abdominal tergites wide, apically rounded, mostly not fused.

##### Description.

**Figure 7.**
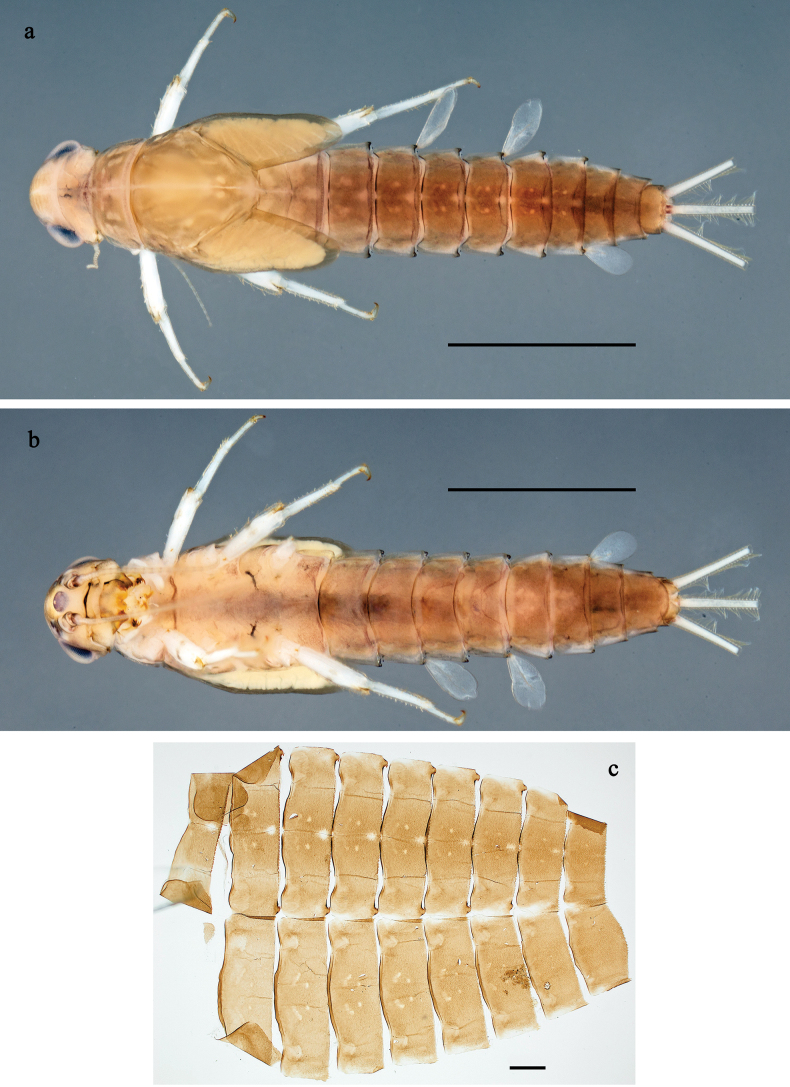
*Labiobaetis tenasserimensis* sp. nov., larva: a. Habitus, dorsal view; b. Habitus, ventral view; c. Abdomen, dorsal and ventral view. Scale bars: 1 mm (a, b); 100 µm (c).

**Figure 8.**
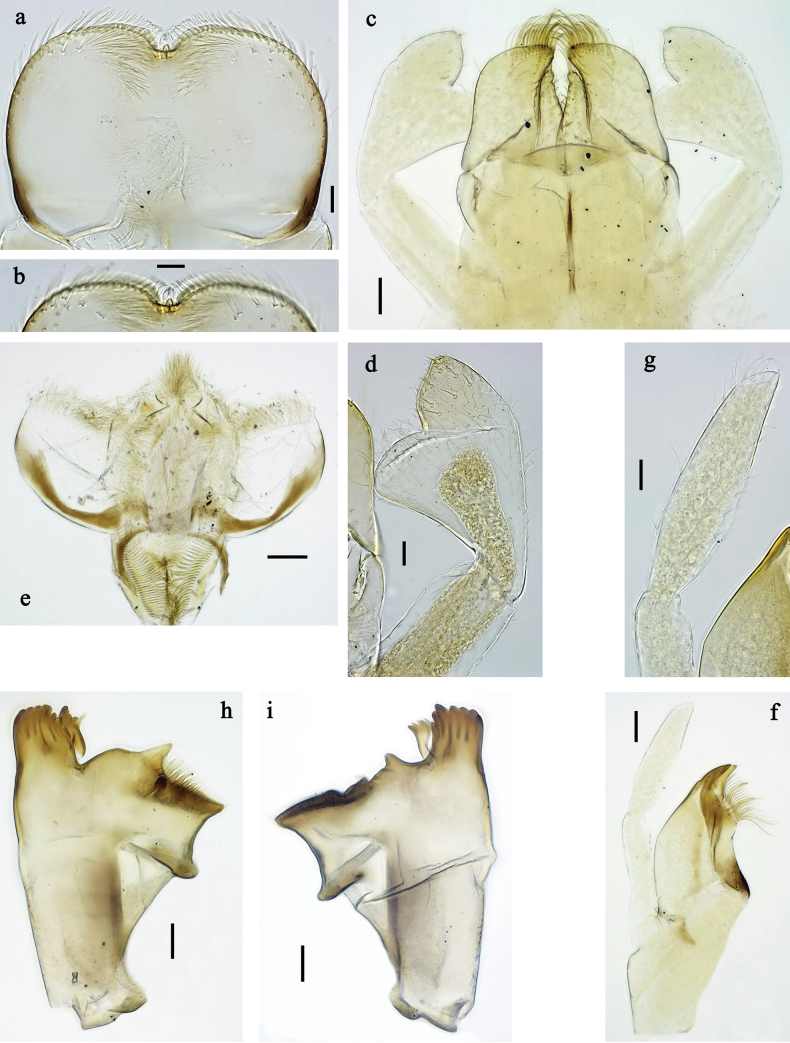
*Labiobaetis tenasserimensis* sp. nov., larva morphology: a. Labrum; b. Section of labrum, dorsal focus; c. Labium; d. Labial palp; e. Hypopharynx and superlinguae; f. Maxilla; g. Maxillary palp; h. Left mandible; i. Right mandible. Scale bars: 20 µm (c, e, f, h, i); 10 µm (a, b, d, g).

**Figure 9.**
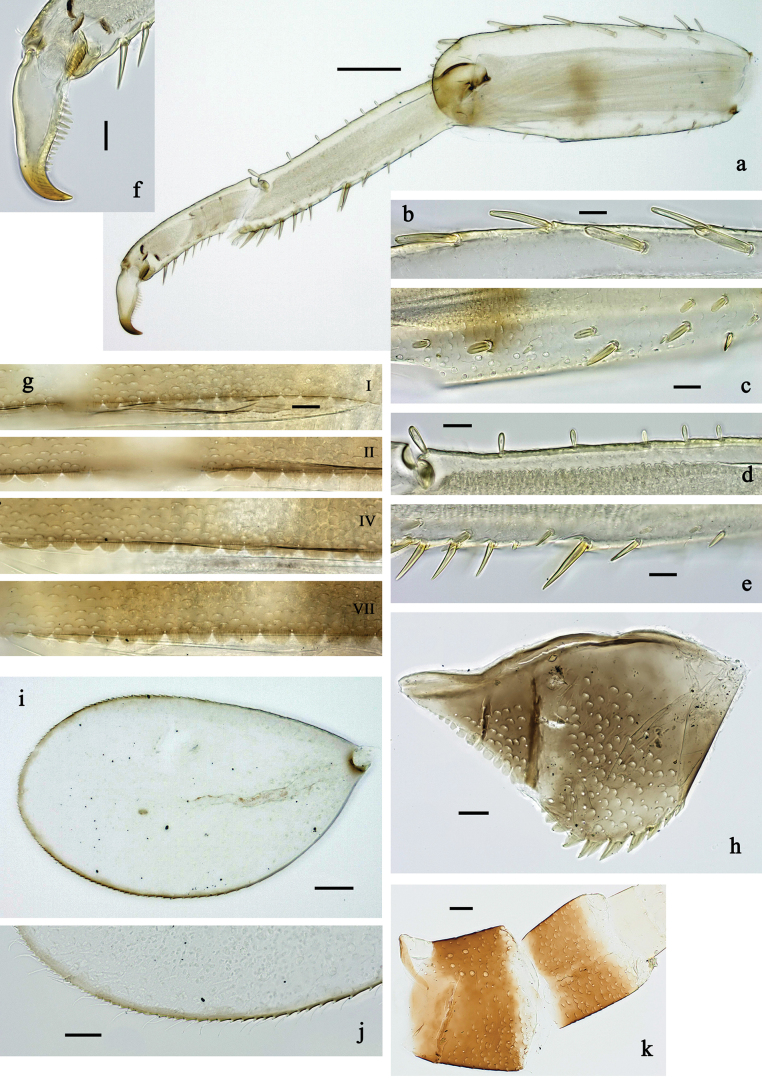
*Labiobaetis tenasserimensis* sp. nov., larva morphology: a. Middle leg; b. Outer margin of middle femur; c. Inner margin of middle femur; d. Outer margin of middle tibia; e. Inner margin of middle tibia; f. Middle claw; g. Posterior margins of abdominal terga; h. Paraproct; i, j. Tergalius IV; k. Antennal base. Scale bars: 50 µm (a); 20 µm (i); 10 µm (b–h, j, k).

**Larva** (Figs 7–9). Body length ~3.5 mm. Cerci ~1/2× body length; paracercus ~0.4× body length. Antenna: ~2× as long as head length.

***Colouration*** (Fig. 7a–c). Head and thorax dorsally beige, abdomen dorsally rather uniform brown. Fore protoptera beige with paler striation. Head and thorax ventrally beige, abdomen ventrally brown. Legs off-white to pale grey-brown, femur medially slightly darker. Caudalii pale grey-brown with dark brown band in distal 1/2.

***Antenna*** (Fig. 9k) with scape and pedicel sub cylindrical, distolateral process at scape absent.

***Labrum*** (Fig. 8a, b). Sub-rectangular, length 0.7× maximum width. Distal margin with medial emargination and small process. Dorsally with medium, fine, simple setae scattered over surface; pair of submedian setae, and submarginal arc of four or five long, simple setae on each side, 1^st^ and 2^nd^ setae closely together. Ventrally with marginal row of setae composed of lateral and anterolateral long, feathered setae and medial long, bifid setae.

***Right mandible*** (Fig. 8i). Incisor and kinetodontium fused. Incisor with four denticles; kinetodontium with three denticles, inner margin of innermost denticle with row of thin setae. Prostheca robust, apically denticulate. Margin between prostheca and mola with pronounced hump. Tuft of setae on proximal corner of mola present. Fine setae scattered along basal margin of mola.

***Left mandible*** (Fig. 8h). Incisor and kinetodontium fused. Incisor with four denticles, kinetodontium with three denticles. Prostheca robust, apicolaterally with small denticles and comb-shaped structure. Margin between prostheca and mola convex, smooth. Tuft of setae on proximal corner of mola present.

Both mandibles with lateral margins almost straight.

***Hypopharynx and superlinguae*** (Fig. 8e). Lingua longer than superlinguae. Lingua longer than broad; medial tuft of stout setae well developed. Superlinguae with lateral margins rounded; fine, long, simple setae along distal margin; strongly sclerotised along laterobasal margin.

***Maxilla*** (Fig. 8f, g). Galea-lacinia ventrally with two simple, apical setae below canines. Medially with one feathered, spine-like seta and two or three medium to long simple setae. Maxillary palp longer than length of galea-lacinia; 2-segmented; palp segment II approx. as long as segment I; setae on maxillary palp fine, simple, scattered over surface of segments I and II; apex of last segment without distolateral excavation, apically slightly pointed.

***Labium*** (Fig. 8c, d). Glossa basally broad, narrowing toward apex; shorter than paraglossa; inner margin with ~8 robust, spine-like seta, distalmost seta much longer; apex with two long and one medium, robust, apically pectinate setae, and one short seta; outer margin with ~4 spine-like setae; ventral surface with fine, simple, scattered setae. Paraglossa sub-rectangular, slightly curved inward; apex rounded; with three rows of long, robust, distally pectinate setae in apical area and two short, simple setae in anteromedial area; dorsally with five long, spine-like setae near inner margin. Labial palp with segment I ~0.9× length of segments II and III combined. Segment II with lobed, thumb-like, distomedial protuberance; distomedial protuberance 0.8× width of base of segment III; dorsally with row of 4–6 spine-like setae near outer margin. Segment III slightly pentagonal; length ~0.8× maximal width; ventrally covered with short, spine-like, simple setae and short, fine, simple setae.

***Hind protoptera*** vestigial.

***Legs*** (Fig. 9a–f). Ratio of foreleg segments 1.4:1.0:0.8:0.3, middle leg 1.3:1.0:0.7:0.3, hind leg 1.4:1.0:0.6:0.3. ***Femur***. Femur length ~3× maximum width. Outer margin with row of five or six spine-like setae and submarginally partial 2^nd^ row; length of setae ~0.2× maximum width of femur. Apex rounded, with pair of spine-like setae and short, stout, apically blunt setae. Stout, lanceolate, pointed setae scattered along inner margin; femoral patch absent or rudimentary on all legs. ***Tibia.*** Outer margin with row of short, stout, apically rounded setae, distalmost seta larger. Inner margin with two rows of medium spine-like setae; on apex tuft of fine, simple setae. Patella-tibial suture present on basal 1/2. ***Tarsus.*** Outer margin almost bare. Inner margin with row of curved, spine-like setae increasing in length distally. ***Claw*** with one row of ~14 denticles; distally pointed.

***Abdominal terga*** (Fig. 9g). Surface with irregular rows of U-shaped scale bases and fine, simple, scattered setae. Posterior margin of terga: I–IX with wide, apically rounded spines, mostly not fused with each other; VI–IX more subtriangular, apically rounded.

***Abdominal sterna*** (Fig. 7c). Posterior margin of sterna: I–V smooth, without spines; VI–IX with triangular, apically rounded spines, similar to spines on tergites.

***Tergalii*** (Fig. 9i, j). Present on segments II–VII. Margin with small denticles intercalating fine, simple setae. Tracheae limited to main trunk. Tergalius IV as long as segments V and 1/2 VI combined; tergalius VII as long as segments VIII and 1/2 IX combined.

***Paraproct*** (Fig. 9h). Distally not expanded, with ~11 stout, marginal spines. Surface scattered with U-shaped scale bases and fine, simple setae. Cercotractor with numerous small, marginal spines.

##### Imago.

Unknown.

##### Comparison.

The most similar species is *L. paranumeratus* Kaltenbach & Gattolliat, 2019 from Sumatra. The following characters distinguish the larvae of both species (see Kaltenbach and Gattolliat 2019b: 62, figs 31, 32): dorsal colour pattern of larval abdomen rather uniform brown in *L. tenasserimensis* sp. nov. (brown with segment V dark reddish-brown and segment II with dark reddish-brown streaks in *L. paranumeratus*); spines on posterior margins of abdominal tergites wide, rounded, mostly with small gaps between them in *L. tenasserimensis* sp. nov. (wide, rounded and mostly fused in *L. paranumeratus*); hypopharynx with medial tuft well developed in *L. tenasserimensis* sp. nov. (poorly developed in *L. paranumeratus*); setae at outer margin of tibia apically rounded, smooth in *L. tenasserimensis* sp. nov. (apically rounded and with minute serration in *L. paranumeratus*).

##### Etymology.

The species name is derived from the name of the Tenasserim mountain range where it was collected.

##### Distribution.

Thailand (Fig. 32b).

#### ﻿ Labiobaetis angularis sp. nov.

Taxon classificationAnimaliaEphemeropteraBaetidae

5EA0CE53-7668-5274-83F5-4A58460F6DEF

https://zoobank.org/63BEC25D-40D6-4803-8B9C-ECC29B29FA5A

Figs 10, 11, 12

##### Type material.

***Holotype*.** Thailand • larva; Petchburi Prov., Kaeng Krachan Distr., Huai Mae Kamoei; 12°58'42"N, 99°34'55"E; 119 m; 24.ii.2018; leg. C. Suttinun; on slide; GBIFCH01223079; VMCMU. ***Paratypes***. 13 larvae; same data as holotype; 2 on slides; GBIFCH00829290, GBIFCH00829279; MZL; 11 in alcohol; GBIFCH00763867; VMCMU • 4 larvae; Kanchanaburi Prov., Thong Pha Phum Distr., Pra Chum Mai; 14°34'58"N, 98°34'52"E; 269 m; 25.v.2017; leg. C. Suttinun; 1 on slide; GBIFCH01223081; VMCMU; 3 in alcohol; GBIFCH00829284, GBIFCH00763860; VMCMU • 2 larvae; Ratchaburi Prov., Suan Pheung Distr., Kang Som Maew; 13°24'22"N, 99°16'44"E; 207 m; 24.xi.2018; leg. C. Suttinun; 1 on slide; GBIFCH00829278; MZL; 1 in alcohol; GBIFCH00829291; VMCMU • 1 larva; Prachuab Khiri Khan Prov., Kui Buri Dist., Huai Sam Rong; 100 m; 12°03'50"N, 99°37'39"E; 19.iv.2019; leg. C. Suttinun; in alcohol; GBIFCH00829287; VMCMU • 6 larvae; Petchburi Prov., Kaeng Krachan Distr., Huai Sat Yai; 12°38'14"N, 99°30'59"E; 162 m; 25.ii.2018; leg. C. Suttinun; in alcohol; GBIFCH00829277, GBIFCH00763858; VMCMU • 7 larvae; Petchburi Prov., Kaeng Krachan Distr., Huai Mae Priang; 12°46'03"N, 99°34'54"E; 142 m; 24.ii.2018; leg. C. Suttinun; in alcohol; GBIFCH00829280, GBIFCH00829274; VMCMU; GBIFCH00763859; MZL • 8 larvae; Ratchaburi Prov., Suan Pheung Distr., Pha Wo Tai; 13°30'57"N, 99°20'40"E; 118 m; 25.xi.2018; leg. C. Suttinun; in alcohol; GBIFCH00829283, GBIFCH00763854, GBIFCH00763861; VMCMU • 4 larvae; Ranong Prov., Mueang Distr., Klong Nok Ngang; 09°42'22"N, 98°34'39"E; 11 m; 21.vi.2018; leg. C. Suttinun; in alcohol; GBIFCH00829273, GBIFCH00763856; VMCMU • 7 larvae; Ratchaburi Prov., Suan Pheung Distr., Swiss valley; 13°30'33"N, 99°17'12"E; 140 m; 26.v.2017; leg. C. Suttinun; in alcohol; GBIFCH00829282, GBIFCH00763857, GBIFCH00763865, GBIFCH00763866; MZL • 1 larva; Ratchaburi Prov., Suan Pheung Distr., Bo Wee resort; 13°28'41"N, 99°14'55"E; 184 m; 24.xi.2018; leg. C. Suttinun; in alcohol; GBIFCH00829289; VMCMU • 2 larvae; Loei Prov., Wang Saphung Distr., Ban Nam Thob; 17°16'11"N, 101°35'51"E; 318 m; 17.xii.2018; leg. C. Suttinun; in alcohol; GBIFCH00829281, GBIFCH00763862; MZL • 3 larvae; Chanthaburi Prov., Khao Khitchakut Distr., Klong Krasue Yai; 12°52'36"N, 102°05'48"E; 38 m; 05.vi.2018; leg. C. Suttinun; in alcohol; GBIFCH00829276, GBIFCH00763864; VMCMU • 1 larva; Prachuap Khiri Khan Prov., Kui Buri Distr., Huai Sam Rong; 12°03'82"N, 99°37'64"E; 103 m; 19.iv.2019; leg. C. Suttinun; on slide; GBIFCH00596153; VMCMU • 1 larva; Prachuap Khiri Khan Prov., Kui Buri Distr., Klong Kui; 12°08'17"N, 99°39'77"E; 104 m; 19.iv.2019; leg. C. Suttinun; on slide; GBIFCH01223080; VMCMU.

##### Diagnosis.

**Larva.** Following combination of characters differentiate *L. angularis* sp. nov. from other species of the group *numeratus*: abdomen dorsally dark grey-brown, laterally off-white; left mandible with angular hump at margin between prostheca and mola; claw with 14–17 denticles; spines at posterior margin of abdominal tergites wide, apically rounded.

##### Description.

**Figure 10.**
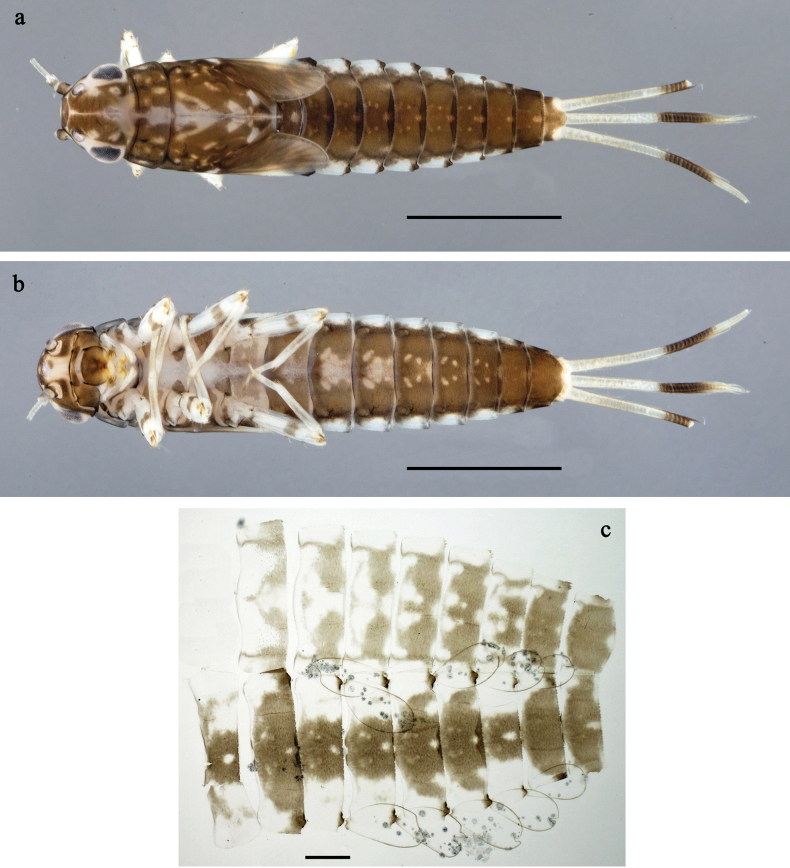
*Labiobaetis angularis* sp. nov., larva: a. Habitus, dorsal view; b. Habitus, ventral view; c. Abdomen, dorsal and ventral view. Scale bars: 1 mm (a, b); 100 µm (c).

**Figure 11.**
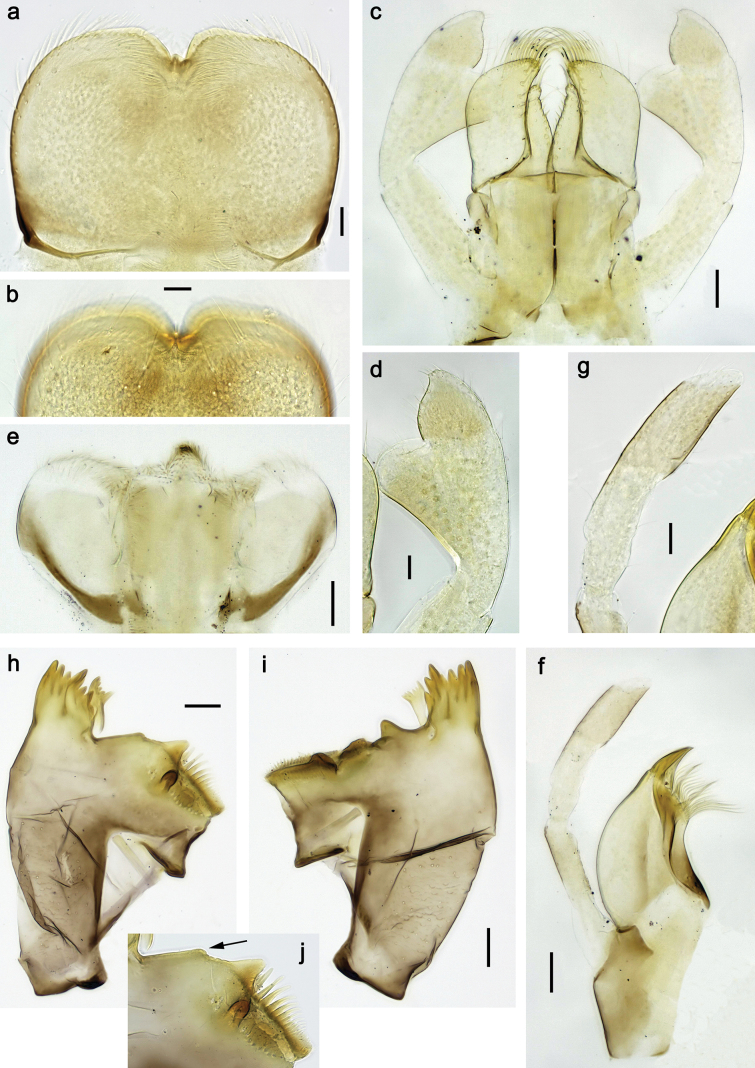
*Labiobaetis angularis* sp. nov., larva morphology: a. Labrum; b. Section of labrum, dorsal focus; c. Labium; d. Labial palp; e. Hypopharynx and superlinguae; f. Maxilla; g. Maxillary palp; h. Left mandible; i. Right mandible; j. Section of left mandible. Scale bars: 20 µm (c, e, f, h, i); 10 µm (a, b, d, g).

**Figure 12.**
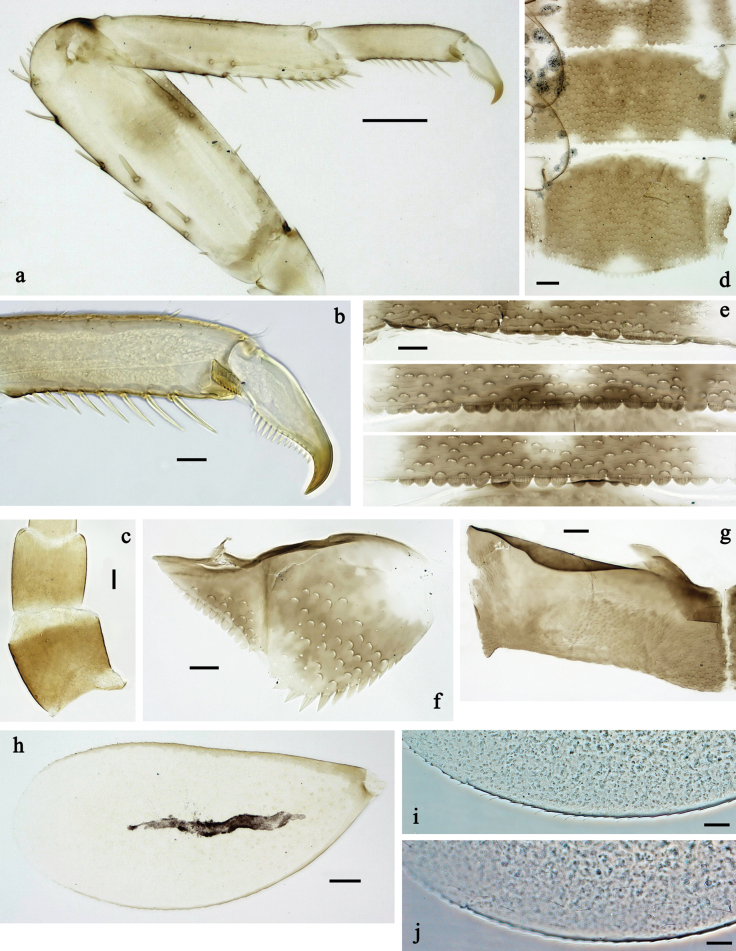
*Labiobaetis angularis* sp. nov., larva morphology: a. Middle leg; b. Middle tarsus and claw; c. Antennal base; d. Abdominal sterna VII–IX; e. Posterior margins of abdominal terga I, V, VIII; f. Paraproct; g. Left half of metanotum with vestigial hind protopteron; h–j. Tergalius IV. Scale bars: 50 µm (a); 20 µm (g, h); 10 µm (b–f, h–j).

**Larva** (Figs 10–12). Body length ~3.3 mm. Cerci ~1/2× body length; paracercus ~0.4× body length. Antenna: ~2× as long as head length.

***Colouration*** (Fig. 10a–c). Head and thorax dorsally dark grey-brown with grey-brown and grey markings; abdomen dorsally dark grey-brown, segment X paler, segments I–VIII laterally off-white. Fore protoptera dark grey and grey-brown. Head and thorax ventrally mainly grey; abdomen ventrally dark grey-brown, segments I and X paler, segments II–V medially paler, segments I–VIII laterally off-white. Legs off-white, femur distomedially with grey-brown band, tibia with long grey-brown streak. Caudalii pale grey with dark grey-brown band in distal 1/2.

***Antenna*** (Fig. 12c) with scape and pedicel sub cylindrical, distolateral process at scape absent.

***Labrum*** (Fig. 11a, b). Sub-rectangular, length 0.7× maximum width. Distal margin with medial emargination and small process. Dorsally with medium, fine, simple setae scattered over surface; pair of submedian setae, and submarginal arc of ~7 long, simple setae on each side, 1^st^ and 2^nd^ setae closely together. Ventrally with marginal row of setae composed of lateral and anterolateral long, feathered setae and medial long, bifid setae.

***Right mandible*** (Fig. 11i). Incisor and kinetodontium fused. Incisor with four denticles; kinetodontium with four denticles, inner margin of innermost denticle with row of thin setae. Prostheca robust, apically denticulate. Margin between prostheca and mola with pronounced hump. Tuft of setae on proximal corner of mola present. Fine setae scattered along basal margin of mola.

***Left mandible*** (Fig. 11h). Incisor and kinetodontium fused. Incisor with four denticles, kinetodontium with three denticles. Prostheca robust, apicolaterally with small denticles and comb-shaped structure. Margin between prostheca and mola with angular hump. Tuft of setae on proximal corner of mola present.

Both mandibles with lateral margins slightly convex.

***Hypopharynx and superlinguae*** (Fig. 11e). Lingua approx. as long as superlinguae. Lingua longer than broad; medial tuft of stout setae well developed, short. Superlinguae with lateral margins rounded; fine, long, simple setae along distal margin; strongly sclerotised along laterobasal margin.

***Maxilla*** (Fig. 11f, g). Galea-lacinia ventrally with two simple, apical setae below canines. Medially with one feathered, spine-like seta and three or four medium to long simple setae. Maxillary palp longer than length of galea-lacinia; 2-segmented; palp segment II ~1.2× length of segment I; setae on maxillary palp fine, simple, scattered over surface of segments I and II; apex of last segment without distolateral excavation, apically slightly pointed.

***Labium*** (Fig. 11c, d). Glossa basally broad, narrowing toward apex; shorter than paraglossa; inner margin with 9–11 robust, spine-like seta, distalmost seta much longer; apex with three long, robust, apically pectinate setae; outer margin with ~4 spine-like setae; ventral surface with fine, simple, scattered setae. Paraglossa sub-rectangular, slightly curved inward; apex rounded; with three rows of long, robust, distally pectinate setae in apical area and ~3 medium, simple setae in anteromedial area; dorsally with six long, spine-like setae near inner margin. Labial palp with segment I ~0.9× length of segments II and III combined. Segment II with lobed, thumb-like, distomedial protuberance; distomedial protuberance 0.9× width of base of segment III; dorsally with row of ~5 spine-like setae near outer margin. Segment III slightly pentagonal, apically truncate; length ~1.1× maximal width; ventrally covered with short, spine-like, simple setae and short, fine, simple setae.

***Hind protoptera*** (Fig. 12g) vestigial.

***Legs*** (Fig. 12a, b). Ratio of foreleg segments 1.3:1.0:0.6:0.3, middle leg 1.3:1.0:0.7:0.3, hind leg 1.4:1.0:0.8:0.3. ***Femur***. Femur length ~2.7× maximum width. Outer margin with row of 4–6 spine-like setae and submarginally partial 2^nd^ row; length of setae ~0.27× maximum width of femur. Apex rounded, with pair of spine-like setae and short, stout, apically blunt setae. Stout, lanceolate, pointed setae scattered along inner margin; femoral patch absent on all legs. ***Tibia.*** Outer margin with row of short, stout, apically rounded setae, sometimes almost bare, distalmost seta larger. Inner margin with two rows of medium spine-like setae; on apex tuft of fine, simple setae. Patella-tibial suture present on basal 1/2. ***Tarsus.*** Outer margin with row of short, apically rounded setae, sometimes almost bare. Inner margin with row of curved, spine-like setae increasing in length distally. ***Claw*** with one row of 14–17 denticles; distally pointed.

***Abdominal terga*** (Fig. 12e). Surface with irregular rows of U-shaped scale bases and fine, simple, scattered setae. Posterior margin of terga: I–IX with wide, apically rounded spines, partly fused with each other.

***Abdominal sterna*** (Fig. 12d). Posterior margin of sterna: I–VI smooth, without spines; VII–IX with triangular spines.

***Tergalii*** (Fig. 12h– j). Present on segments II–VII. Margin with small denticles intercalating fine, simple setae. Tracheae limited to main trunk. Tergalius IV as long as segments V and VI combined; tergalius VII as long as segments VIII and 1/2 IX combined.

***Paraproct*** (Fig. 12f). Distally not expanded, with ~13 stout, marginal spines. Surface scattered with U-shaped scale bases and fine, simple setae. Cercotractor with numerous small, marginal spines.

##### Imago.

Unknown.

##### Etymology.

The Latin word *angularis*, meaning angular, refers to the unique angular hump at the margin between prostheca and mola of the left mandible.

##### Distribution.

Thailand (Fig. 32b).

#### ﻿ Labiobaetis tonsator sp. nov.

Taxon classificationAnimaliaEphemeropteraBaetidae

A595384F-74B3-5638-A344-DFCEB1779AB6

https://zoobank.org/641AA02F-1D43-4B1C-A0FD-A17D57334A4D

Figs 13, 14, 15

##### Type material.

***Holotype*.** Thailand • larva; Patthalung Prov., Ton Sa Tor; 07°11'43"N, 100°04'18"E; 51 m; 12.iii.2016; leg. C. Suttinun; on slide; GBIFCH00829272; VMCMU. ***Paratype*.** 1 larva; same data as holotype; on slide; GBIFCH00763863; MZL.

##### Diagnosis.

**Larva.** Following combination of characters differentiate *L. tonsator* sp. nov. from other species of the group *numeratus*: abdomen dorsally grey-brown, segment II distomedially with dark grey-brown spot, segment V dark brown; pedicellus basally with dark brown hypodermal colouration; claw with 12–16 denticles; spines at posterior margin of abdominal tergites wide, apically rounded, mostly not fused.

##### Description.

**Figure 13.**
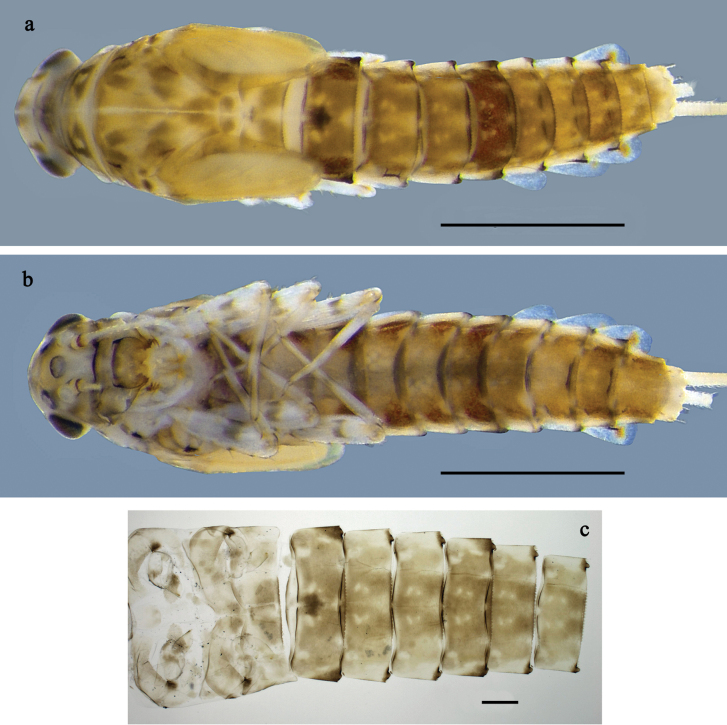
*Labiobaetis tonsator* sp. nov., larva: a. Habitus, dorsal view; b. Habitus, ventral view; c. Abdomen, dorsal view. Scale bars: 1 mm (a, b); 100 µm (c).

**Figure 14.**
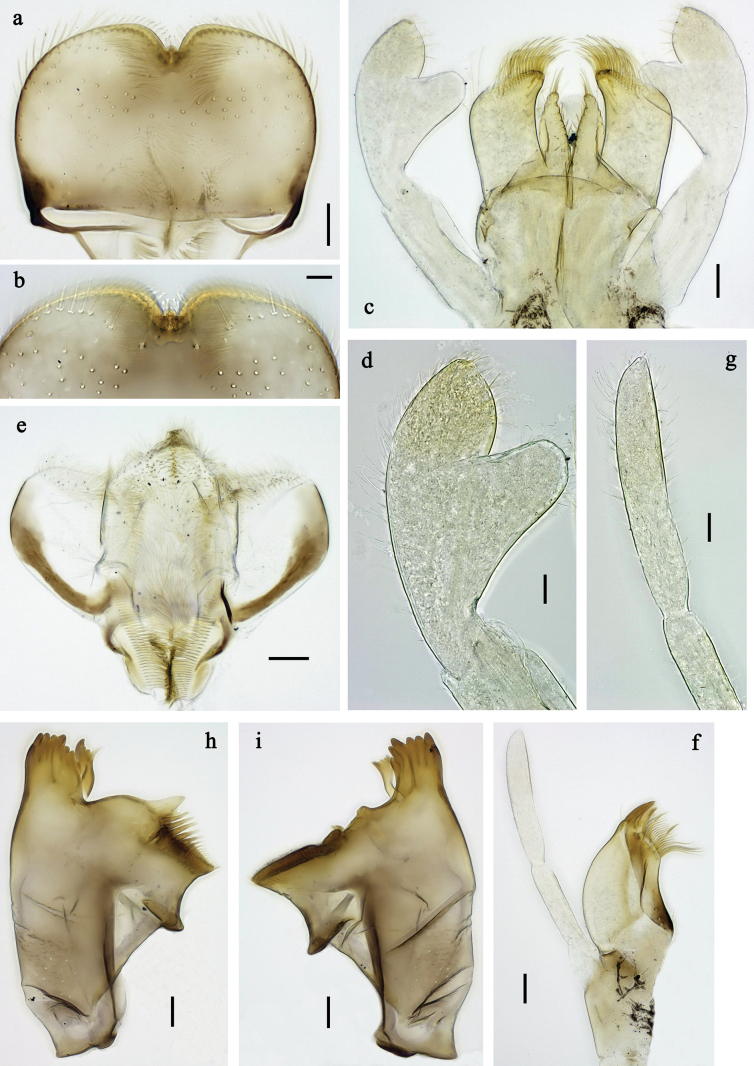
*Labiobaetis tonsator* sp. nov., larva morphology: a. Labrum; b. Section of labrum, dorsal focus; c. Labium; d. Labial palp; e. Hypopharynx and superlinguae; f. Maxilla; g. Maxillary palp; h. Left mandible; i. Right mandible. Scale bars: 20 µm (c, e, f, h, i); 10 µm (a, b, d, g).

**Figure 15.**
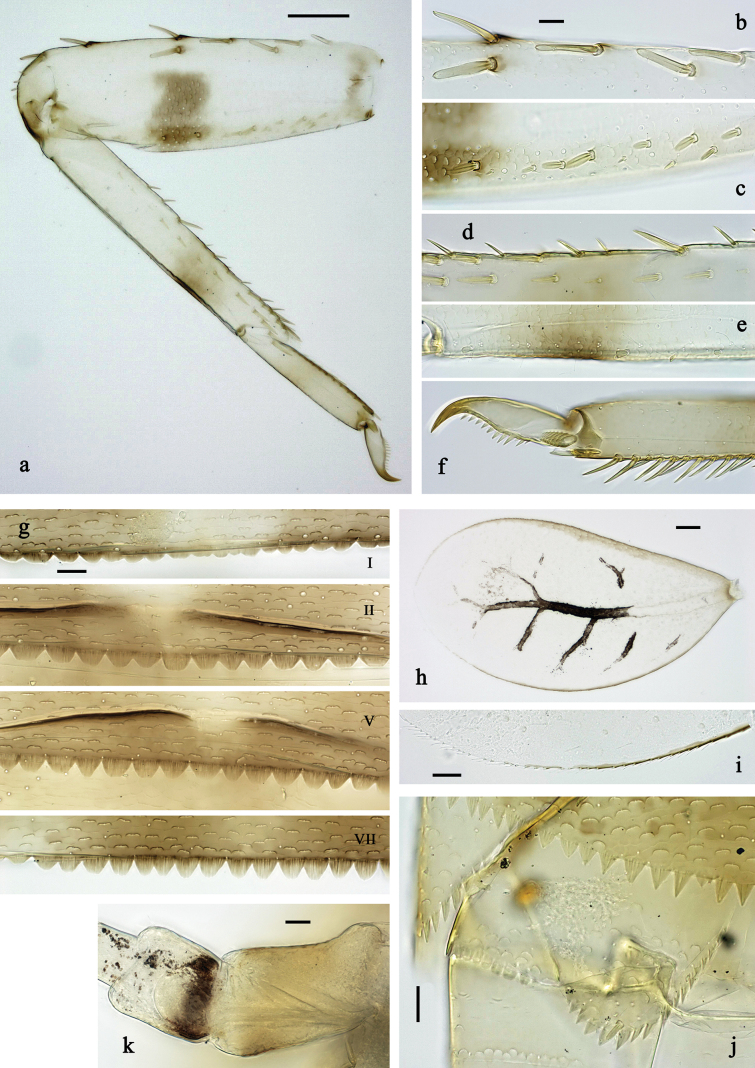
*Labiobaetis tonsator* sp. nov., larva morphology: a. Middle leg; b. Outer margin of middle femur; c. Inner margin of middle femur; d. Inner margin of middle tibia; e. Outer margin of middle tibia; f. Fore tarsus and claw; g. Posterior margins of abdominal terga; h, i. Tergalius III; j. Paraproct; k. Antennal base. Scale bars: 50 µm (a); 20 µm (h); 10 µm (b–g, i–k).

**Larva** (Figs 13–15). Body length 3.3–3.6 mm. Caudalii: broken. Antenna: ~2× as long as head length.

***Colouration*** (Fig. 13a–c). Head and thorax dorsally yellow-grey with grey markings as in Fig. 13a, particularly with grey, round dot at base of fore protoptera; abdomen dorsally grey-brown, laterally yellow-grey, segment X paler, segment V dark brown, segment II distomedially with dark grey-brown, roundish spot, laterally dark grey-brown. Fore protoptera yellow-grey. Head, thorax and abdomen ventrally mainly dark grey, segment X paler, abdomen laterally yellow-grey. Legs pale grey, femur distomedially with grey and yellow markings, tibia distomedially with grey area. Caudalii whitish-yellow.

***Antenna*** (Fig. 15k) with scape and pedicel sub cylindrical, distolateral process at scape absent.

***Labrum*** (Fig. 14a, b). Sub-rectangular, length 0.7× maximum width. Distal margin with medial emargination and small process. Dorsally with medium, fine, simple setae scattered over surface; pair of submedian setae, and submarginal arc of ~9 long, simple setae on each side, 1^st^ and 2^nd^ setae closely together. Ventrally with marginal row of setae composed of lateral and anterolateral long, feathered setae and medial long, bifid setae.

***Right mandible*** (Fig. 14i). Incisor and kinetodontium fused. Incisor with four denticles; kinetodontium with three denticles, inner margin of innermost denticle with row of thin setae. Prostheca robust, apically denticulate. Margin between prostheca and mola with pronounced hump. Tuft of setae on proximal corner of mola present. Fine setae scattered along basal margin of mola.

***Left mandible*** (Fig. 14h). Incisor and kinetodontium fused. Incisor with four denticles, kinetodontium with three denticles. Prostheca robust, apicolaterally with small denticles and comb-shaped structure. Margin between prostheca and mola slightly convex. Tuft of setae on proximal corner of mola present.

Both mandibles with lateral margins almost straight.

***Hypopharynx and superlinguae*** (Fig. 14e). Lingua slightly longer than superlinguae. Lingua longer than broad; medial tuft of stout setae well developed, short. Superlinguae with lateral margins rounded; fine, long, simple setae along distal margin; strongly sclerotised along laterobasal margin.

***Maxilla*** (Fig. 14f, g). Galea-lacinia ventrally with two simple, apical setae below canines. Medially with one feathered, spine-like seta and four medium to long simple setae. Maxillary palp longer than length of galea-lacinia; 2-segmented; palp segment II approx. as long as segment I; setae on maxillary palp fine, simple, scattered over surface of segments I and II; apex of last segment without distolateral excavation, apically slightly pointed.

***Labium*** (Fig. 14c, d). Glossa basally broad, narrowing toward apex; shorter than paraglossa; inner margin with ~9 robust, spine-like seta, distalmost seta much longer; apex with three long, robust, apically pectinate setae and one short, robust seta; outer margin with ~4 spine-like setae; ventral surface with fine, simple, scattered setae. Paraglossa sub-rectangular, slightly curved inward; apex rounded; with three rows of long, robust, distally pectinate setae in apical area and ~3 medium, simple setae in anteromedial area; dorsally with five or six long, spine-like setae near inner margin. Labial palp with segment I ~0.8× length of segments II and III combined. Segment II with slightly elongated thumb-like, distomedial protuberance; distomedial protuberance 0.8× width of base of segment III; dorsally with row of five spine-like setae near outer margin. Segment III slightly elongate, conical, apically truncate; length approx. equal to maximal width; ventrally covered with short, spine-like, simple setae and short, fine, simple setae.

***Hind protoptera*** vestigial.

***Legs*** (Fig. 15a–f). Ratio of foreleg segments 1.2:1.0:0.6:0.2, middle leg 1.1:1.0:0.5:0.2, hind leg 1.2:1.0:0.5:0.2. ***Femur***. Femur length ~3× maximum width. Outer margin with row of 5–7 spine-like setae and submarginally partial 2^nd^ row; length of setae ~0.27× maximum width of femur. Apex rounded, with pair of spine-like setae and short, stout, apically blunt setae. Stout, lanceolate, pointed setae scattered along inner margin; femoral patch absent on all legs. ***Tibia.*** Outer margin with row of few short, stout, apically blunt setae, distalmost seta larger. Inner margin with two rows of medium spine-like setae; on apex tuft of fine, simple setae. Patella-tibial suture present on basal 1/2. ***Tarsus.*** Outer margin with row of short, apically blunt setae, sometimes almost bare. Inner margin with row of curved, spine-like setae increasing in length distally. ***Claw*** with one row of 12–16 denticles; distally pointed.

***Abdominal terga*** (Fig. 15e). Surface with irregular rows of U-shaped scale bases and fine, simple, scattered setae. Posterior margin of terga: I–IX with wide, toward end of abdomen subtriangular, apically rounded spines, rarely fused with each other.

***Abdominal sterna***. Posterior margin of sterna: I–VI smooth, without spines; VII–IX with wide triangular spines.

***Tergalii*** (Fig. 15h, i). Present on segments II–VII. Margin with small denticles intercalating fine, simple setae. Tracheae extending from main trunk to inner and outer margins. Tergalius IV as long as segments V and 1/2 VI combined; tergalius VII as long as segments VIII and 1/2 IX combined.

***Paraproct*** (Fig. 15j). Distally not expanded, with ~17 stout, marginal spines. Surface scattered with U-shaped scale bases and fine, simple setae. Cercotractor with numerous small, marginal spines.

##### Imago.

Unknown.

##### Etymology.

The species name is derived from the name of the village Ton Sa Tor, where it was collected (type locality).

##### Distribution.

Thailand (Fig. 32b).

### ﻿*Labiobaetis operosus* species group (Kaltenbach and Gattolliat 2019b: 71)

**Diagnosis**. Labrum dorsal surface with submarginal row of feathered setae; labial palp segment II with large, thumb-like protuberance; antennal scape with well-developed distolateral process; hind protoptera well developed; tergalii present on abdominal segments I–VII.

The following species are known from continental Southeast Asia: *Labiobaetis operosus* (Müller-Liebenau, 1984), Labiobaetis cf. paraoperosus Kaltenbach & Gattolliat, 2018, *Labiobaetis brao* Kaltenbach, Garces & Gattolliat, 2022, *Labiobaetis nisaratae* sp. nov.

#### ﻿ Labiobaetis cf. paraoperosus

Taxon classificationAnimaliaEphemeropteraBaetidae

Kaltenbach & Gattolliat, 2018

57CDFA2D-6331-57CD-9148-FF41219C0CAE

##### Material examined.

Thailand • 3 larvae; Ranong Prov., Mueang Distr., Punyaban Waterfall; 10°03'54"N, 98°40'13"E; 52 m; 19.vi.2018; leg. C. Suttinun; on slides; GBIFCH01223074, GBIFCH00596156; VMCMU; GBIFCH00592514; MZL • 9 larvae; Ranong Prov., Mueang Distr., Huai Por Ta Hin Rao; 09°52'08"N, 98°37'32"E; 20 m; 20.vi.2018; leg. C. Suttinun; in alcohol; GBIFCH00763804; VMCMU; GBIFCH00763803; MZL.

##### Diagnosis.

**Larva.** Following combination of characters differentiate *L. paraoperosus* from other species of the group *operosus*: maxillary palp much longer than galea-lacinia, segment II with slight distolateral excavation; labial palp segment II with broad thumb-like, apically rounded distolateral protuberance; labial palp segment III oblong.

##### Remark.

The larva has no morphological difference to *L. paraoperosus* from Indonesia (Sumatra), but there is a genetic distance of 12% (COI, K2P).

##### Distribution.

Thailand (Fig. 32c), but *L. paraoperosus* is known from Sumatra (Indonesia).

#### ﻿ Labiobaetis nisaratae sp. nov.

Taxon classificationAnimaliaEphemeropteraBaetidae

CB7723BB-256C-5280-9C5C-4CF1378F4493

https://zoobank.org/B127C952-7BC2-4E0F-83B0-E530784B585C

Figs 16, 17, 18, 19

##### Type-material.

***Holotype*.** Thailand • larva; Yasothon Prov., Kut Chum Distr., Huai Naso; 15°57'08"N, 104°18'12"E; 125 m; 11.08.2014; leg. B. Boonsoong; on slide; GBIFCH01221811; VMCMU. ***Paratypes*.** 9 larvae, 1 male imago (reared from larva); same data as holotype; 2 larvae on slides; GBIFCH00592508, GBIFCH00592509; MZL; 7 larvae in alcohol; GBIFCH00975862; VMCMU; 1 imago in alcohol; VMCMU • 1 larva; Kanchanaburi Prov., Thong Pha Phum Distr., Pra Chum Mai; 14°34'58"N, 98°34'52"E; 269 m; 25.v.2017; leg. C. Suttinun; on slide; GBIFCH01223084; MZL.

##### Diagnosis.

**Figure 16.**
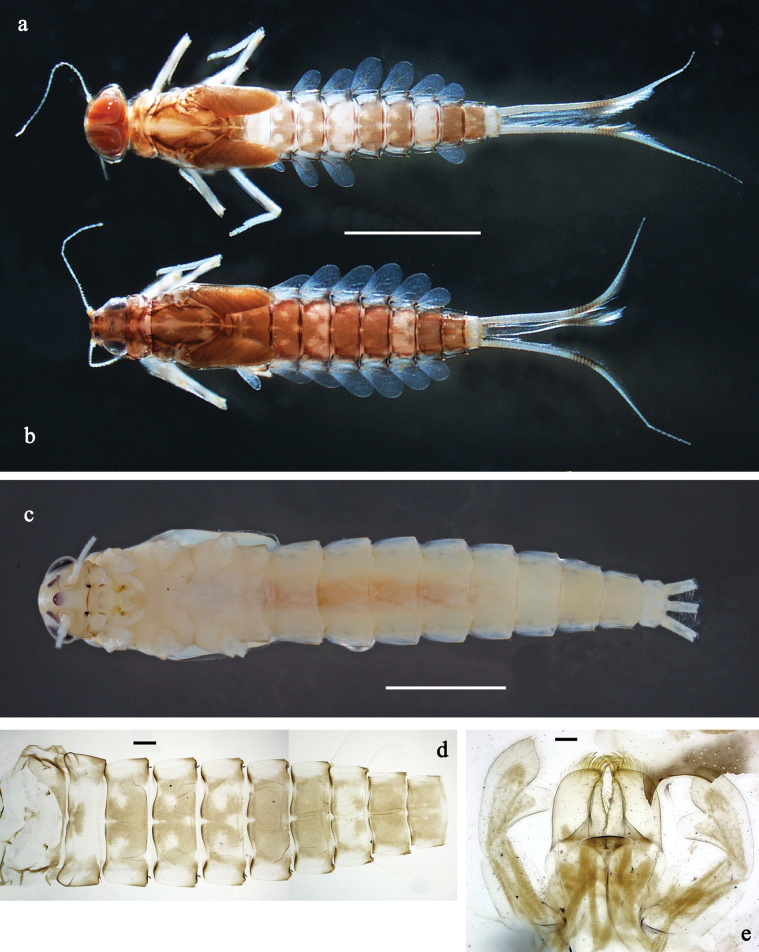
*Labiobaetis nisaratae* sp. nov., larva: a. Habitus male, dorsal view; b. Habitus female, dorsal view; c. Habitus, ventral view; d. Abdomen, dorsal view. *Labiobaetis operosus*, larva, holotype: e. Labium (Malaysia). Scale bars: 1 mm (a–c); 100 µm (d); 20 µm (e).

**Larva.** Following combination of characters differentiate *L. nisaratae* sp. nov. from other species of the group *operosus*: abdomen dorsally brown, segments I–IV with paler areas as in Fig. 16a, b, d, segments VII and X pale; labial palp segment II with elongated, apically rounded, distomedial protuberance, protuberance longer than base of segment III; femoral patch absent on foreleg, well-developed on middle and hind leg.

##### Description.

**Figure 17.**
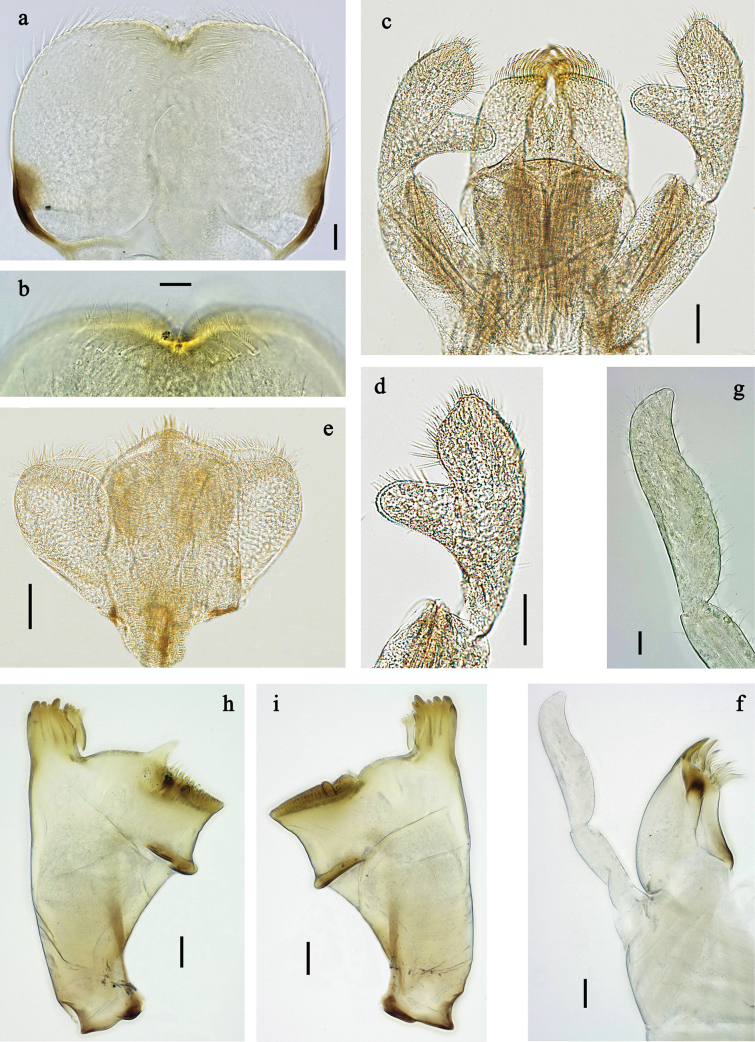
*Labiobaetis nisaratae* sp. nov., larva morphology: a. Labrum; b. Section of labrum, dorsal focus; c. Labium; d. Labial palp; e. Hypopharynx and superlinguae; f. Maxilla; g. Maxillary palp; h. Left mandible i Right mandible. Scale bars: 50 µm (c–e); 20 µm (f, h, i); 10 µm (a, b, g).

**Figure 18.**
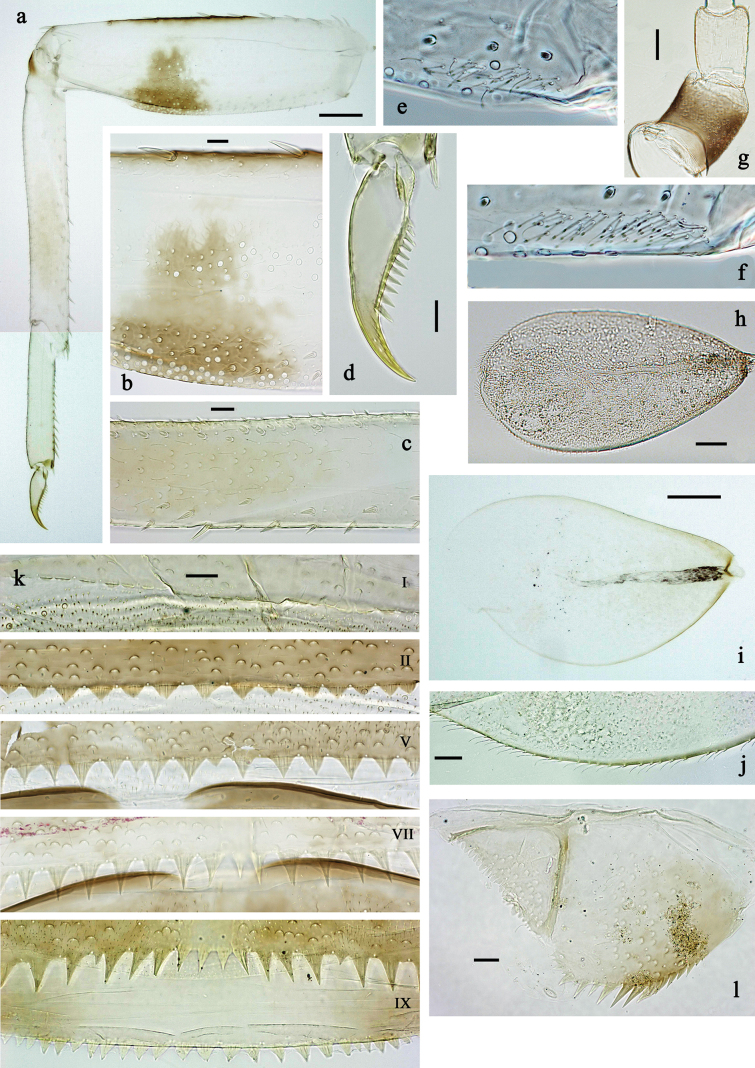
*Labiobaetis nisaratae* sp. nov., larva morphology: a. Fore leg; b. Section of fore femur; c. Section of fore tibia; d. Fore claw; e. Middle leg, femoral patch; f. Hind leg, femoral patch; g. Antennal base; h. Tergalius VII; i, j. Tergalius V; k. Posterior margins of abdominal terga; l. Paraproct. Scale bars: 50 µm (a, g–i); 10 µm (b–f, j–l).

**Larva** (Figs 16–18). Body length 2.9–5.3 mm. Cerci: ~2/3 of body length. Paracercus: ~0.4× body length. Antenna: ~2× as long as head length.

***Colouration*** (Fig. 16a–d). Head and thorax dorsally dark brown with some paler areas; abdomen dorsally brown to dark brown, segments I–IV with paler areas, VII and X pale. Fore protoptera dark brown. Head, thorax and abdomen ventrally beige. Legs off-white to pale brown, femur distomedially with yellow-brown spot. Caudalii off-white to pale brown, with dark brown band in middle part. Cerci distally annulated.

***Antenna*** (Fig. 18g) with scape and pedicel sub cylindrical, distolateral process at scape well developed.

***Labrum*** (Fig. 17a, b). Sub-rectangular, length 0.6× maximum width. Distal margin with medial emargination and small process. Dorsally with medium, fine, simple setae scattered over surface; submarginal arc of ~9 long, feathered setae on each side. Ventrally with marginal row of setae composed of anterolateral long, feathered setae and medial long, bifid setae.

***Right mandible*** (Fig. 17i). Incisor and kinetodontium fused. Incisor with five denticles; kinetodontium with three denticles. Prostheca robust, apically denticulate. Margin between prostheca and mola slightly convex, with few minute denticles. Tuft of setae on proximal corner of mola present.

***Left mandible*** (Fig. 17h). Incisor and kinetodontium fused. Incisor with four denticles, kinetodontium with three denticles. Prostheca robust, apicolaterally with small denticles and comb-shaped structure. Margin between prostheca and mola slightly convex, with minute denticles. Tuft of setae on proximal corner of mola present.

Both mandibles with lateral margins almost straight.

***Hypopharynx and superlinguae*** (Fig. 17e). Lingua slightly longer than superlinguae, longer than broad; medial tuft of stout setae poorly developed. Superlinguae with lateral margins rounded; fine, long, simple setae along distal margin.

***Maxilla*** (Fig. 17f, g). Galea-lacinia ventrally with two simple, apical setae below canines. Medially with one feathered, spine-like seta and three or four medium to long simple setae. Maxillary palp longer than length of galea-lacinia; 2-segmented; palp segment II ~1.2× length of segment I; setae on maxillary palp fine, simple, scattered over surface of segments I and II; apex of last segment with well-developed distolateral excavation.

***Labium*** (Fig. 17c, d). Glossa basally broad, narrowing toward apex; shorter than paraglossa; inner margin with eight or nine robust, spine-like setae, distalmost seta much longer; apex with two long and one medium, robust, apically pectinate setae and one short, robust seta; outer margin with four spine-like setae; ventral surface with fine, simple, scattered setae. Paraglossa sub-rectangular, slightly curved inward; apex rounded; with three rows of long, robust, distally pectinate setae in apical area and four medium, simple setae in medial and anteromedial area; dorsally with four long, spine-like setae near inner margin. Labial palp with segment I approx. as long as segments II and III combined. Segment II with elongated thumb-like, apically rounded, distomedial protuberance; distomedial protuberance 1.1× width of base of segment III; dorsally with row of three or four spine-like setae near outer margin. Segment III slightly pentagonal, apically truncate; length ~1.2× maximal width; ventrally covered with short, spine-like, simple setae and short, fine, simple setae.

**Figure 19.**
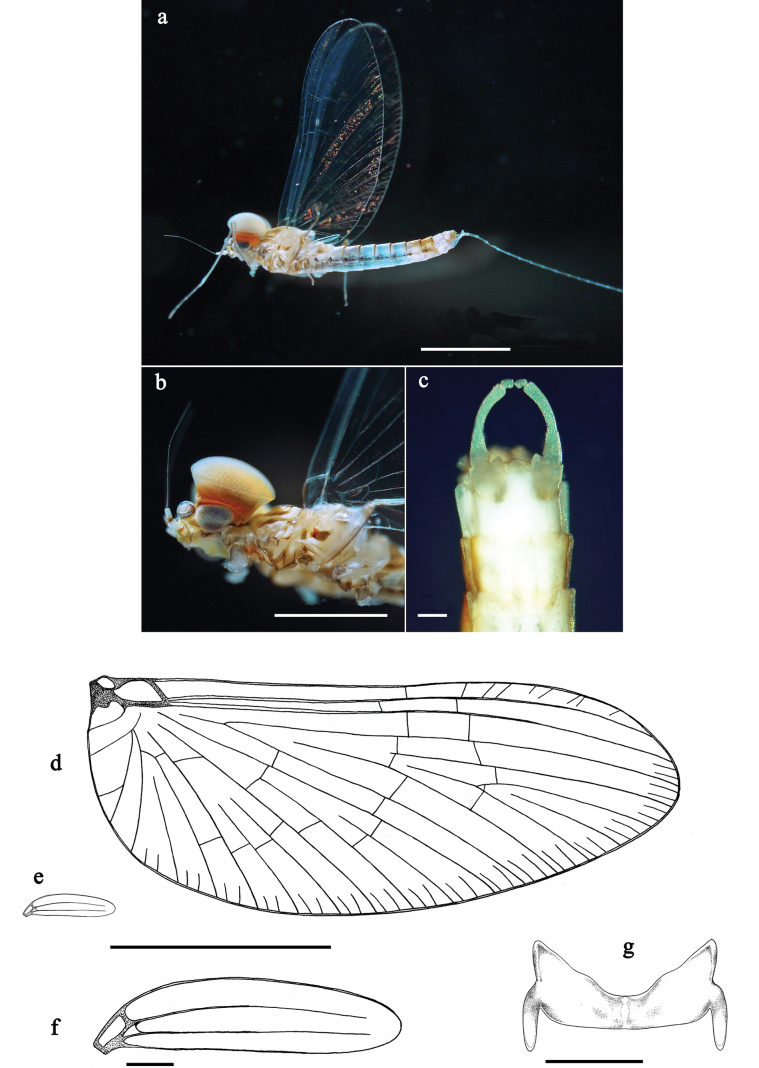
*Labiobaetis nisaratae* sp. nov., male imago: a. Habitus, lateral; b. Head and thorax, lateral; c. Genitalia; d. Fore wing; e. Hind wing (same proportion as d); f. Hind wing (augmented); g. Metanotum with hind protoptera. Scale bars: 1 mm (a, b, d, e); 0.5 mm (g); 100 µm (c, f).

***Hind protoptera*** (Fig. 19g) well developed.

***Legs*** (Fig. 18a–f). Ratio of foreleg segments 1.3:1.0:0.5:0.2, middle leg 1.3:1.0:0.5:0.2, hind leg 1.4:1.0:0.5:0.2. ***Femur***. Femur length ~4× maximum width. Outer margin with row of nine or ten spine-like setae; length of setae ~0.18× maximum width of femur. Apex rounded, with pair of spine-like setae and short, apically blunt setae. Stout, lanceolate, pointed setae scattered along inner margin; femoral patch absent on foreleg and well developed on middle and hind legs. ***Tibia.*** Outer margin with row of many marginal and submarginal, short, apically blunt setae, distalmost seta larger. Inner margin with two rows of medium spine-like setae; on apex tuft of fine, simple setae. Patella-tibial suture present on basal 1/2. ***Tarsus.*** Outer margin with row of short, apically blunt setae. Inner margin with row of curved, short to medium, spine-like setae increasing in length distally. ***Claw*** with one row of 10–12 denticles; distally pointed.

***Abdominal terga*** (Fig. 18k). Surface with irregular rows of U-shaped scale bases and fine, simple, scattered setae. Posterior margin of terga: I with rudimentary spines, II–IX with triangular, pointed spines, becoming longer, narrower and sharper pointed toward end of abdomen; II–IX posterolaterally with one, two, or several minute to small spines, increasing in number toward end of abdomen.

***Abdominal sterna***. Posterior margin of sterna: I–VII smooth, without spines; VIII and IX with triangular spines.

***Tergalii*** (Fig. 18h–j). Present on segments I–VII. Margin with small denticles intercalating fine, simple setae. Tracheae partly extending from main trunk to inner and outer margins. Tergalius I approx. as long as segment II; tergalius IV approx. as long as segments V and VI combined; tergalius VII as long as segments VIII and 1/2 IX combined.

***Paraproct*** (Fig. 18l). Distally not expanded, with ~17 stout, marginal spines. Surface scattered with U-shaped scale bases and fine, simple setae. Cercotractor with numerous small, marginal spines.

**Male imago** (Fig. 19a–f). Body length 2.5 mm, forewing length ~2.5 mm, hind wing length ~0.5 mm.

***Colouration.*** Head and thorax beige with brown markings, turbinate eyes beige, shaft pink. Legs bluish. Wings and venation hyaline. Abdominal segments I–VI dorsally and ventrally bluish, segments VII, IX, and X off-white to beige, segment VIII light brown; abdominal segments dorsally with narrow brown bands at posterior margins. Cerci bluish.

***Forewing***. Pterostigma with seven cross-veins, first two reaching subcostal vein. Double intercalary veins mostly shorter than distance between corresponding main veins at wing margin.

***Hind wing***. Much smaller than forewing, with two longitudinal veins.

***Genitalia.*** Basal segment of gonostylus (unistyliger) distally expanded at inner margin; segments I and II almost completely fused; segment I basally with protuberance at inner margin; small constriction at base of segment II; segment III slightly ovoid, with strong constriction at base, cross-section dimension slightly smaller than distal margin of segment II. Styliger plate between unistyligers well developed, distal margin straight.

##### Comparison.

The larva of *L. nisaratae* sp. nov. is very similar to *L. operosus*. The only clear difference is in the shape of labial palp segment II, where the distomedial protuberance in *L. nisaratae* sp. nov. is elongated, longer than the base of segment III (~1.1×). In *L. operosus*, the distomedial protuberance of segment II is shorter than the base of segment III (~0.8×) and stouter than in *L. nisaratae* sp. nov.

##### Etymology.

The name of this species is dedicated to Assoc. Prof. Dr. Nisarat Tungpairojwong (Department of Biology, Khon Kean University) for her outstanding contributions to the systematics of aquatic insects in Thailand.

##### Distribution.

Thailand (Fig. 32c).

### ﻿*Labiobaetis sumigarensis* species group (Kaltenbach and Gattolliat 2019b: 30)

**Diagnosis**. Labrum dorsal surface with submarginal row of clavate setae, submedian setae absent; left mandible without setae at mola apex; hind protoptera absent; tergalii usually on abdominal segments II–VII; distolateral process at scape absent or poorly developed;

The following species are known from continental Southeast Asia: *Labiobaetis diffundus* (Müller-Liebenau, 1984), *Labiobaetis kui* Kaltenbach, Garces & Gattolliat, 2022, *Labiobaetis karen* sp. nov., *Labiobaetis septem* sp. nov., *Labiobaetis ranongensis* sp. nov.

#### ﻿ Labiobaetis karen sp. nov.

Taxon classificationAnimaliaEphemeropteraBaetidae

1AE9A988-0908-51AE-A41D-1399C93B89F6

https://zoobank.org/69CDB73A-552B-47BF-AABF-DE24C93FD380

Figs 20, 21, 22

##### Type material.

***Holotype*.** Thailand • larva; Kanchanaburi Prov., Thong Pha Phum Distr., Pra Chum Mai; 14°34'58"N, 98°34'52"E; 269 m; 25.v.2017; leg. C. Suttinun; on slide; GBIFCH00763836; VMCMU. ***Paratypes*.** 2 larvae; same data as holotype; on slides; GBIFCH00980859, GBIFCH01221814; MZL • 1 larva; Tak Prov., Mueang Distr., Klong Lan Sang; 16°46'53"N, 99°01'16"E; 253 m; 25.xii.2017; leg. C. Suttinun; on slide; GBIFCH00592515; VMCMU.

##### Diagnosis.

**Larva.** Following combination of characters differentiate *L. karen* sp. nov. from other species of the group *sumigarensis*: thorax dorsally ochre, with dark brown distolateral dots on mesonotum; abdomen dorsally dark reddish-brown, paler in middle area, segment I ochre; femur with dark brown, triangular marking at inner, distomedial margin; tibia dark brown in subdistal area; scapus and pedicellus laterally with dark brown hypodermal colouration; labial palp segment III subrectangular with inner distal margin concave; paraproct distally not expanded.

##### Description.

**Figure 20.**
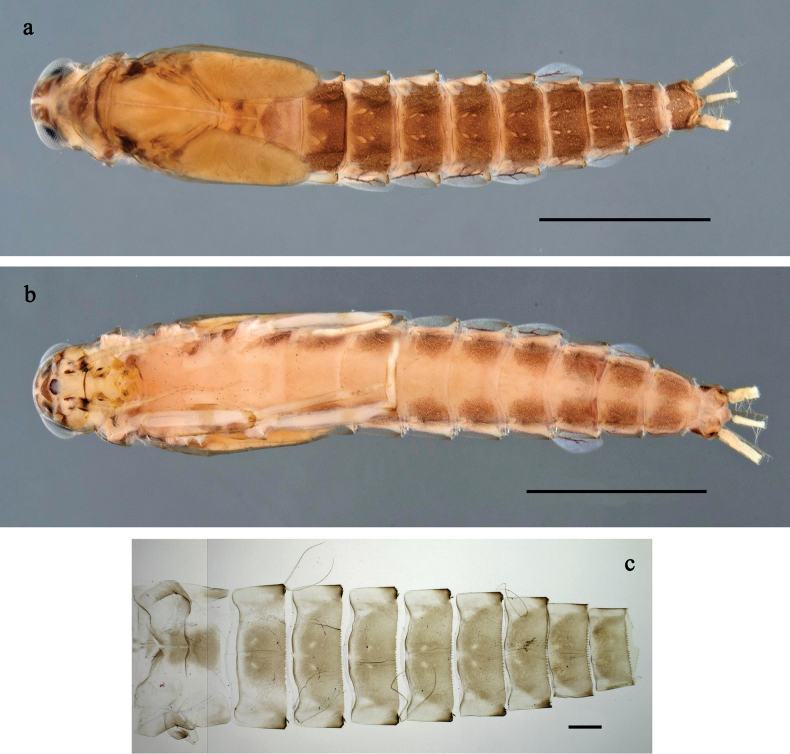
*Labiobaetis karen* sp. nov., larva: a. Habitus, dorsal view; b. Habitus, ventral view; c. Abdomen, dorsal view. Scale bars: 1 mm (a, b); 100 µm (c).

**Figure 21.**
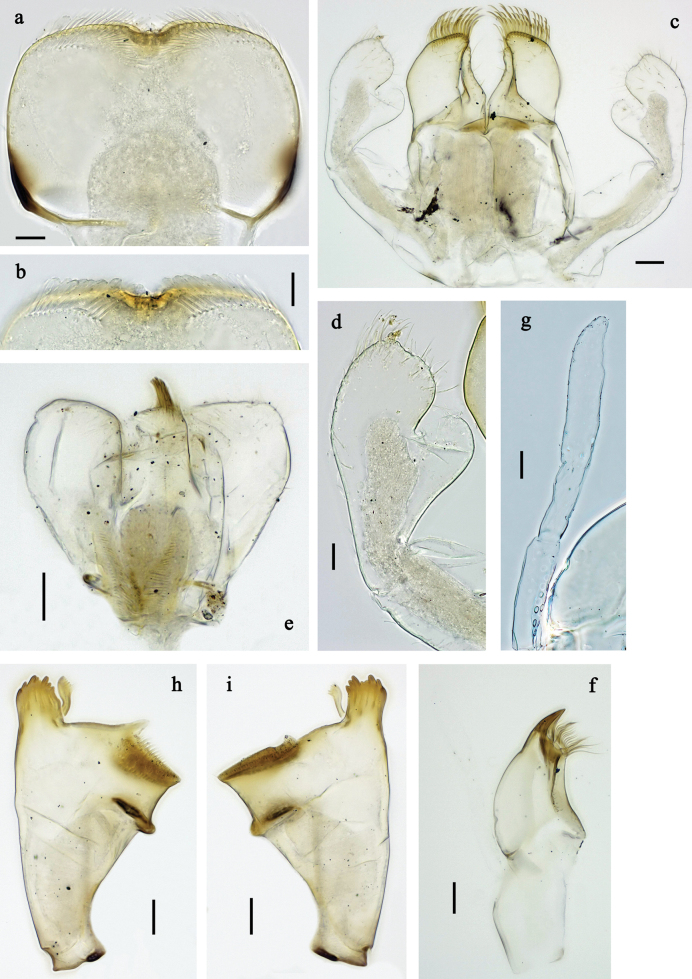
*Labiobaetis karen* sp. nov., larva morphology: a. Labrum; b. Section of labrum, dorsal focus; c. Labium; d. Labial palp; e. Hypopharynx and superlinguae; f. Maxilla; g. Maxillary palp; h. Left mandible; i. Right mandible. Scale bars: 20 µm (c, e, f, h, i); 10 µm (a, b, d, g).

**Figure 22.**
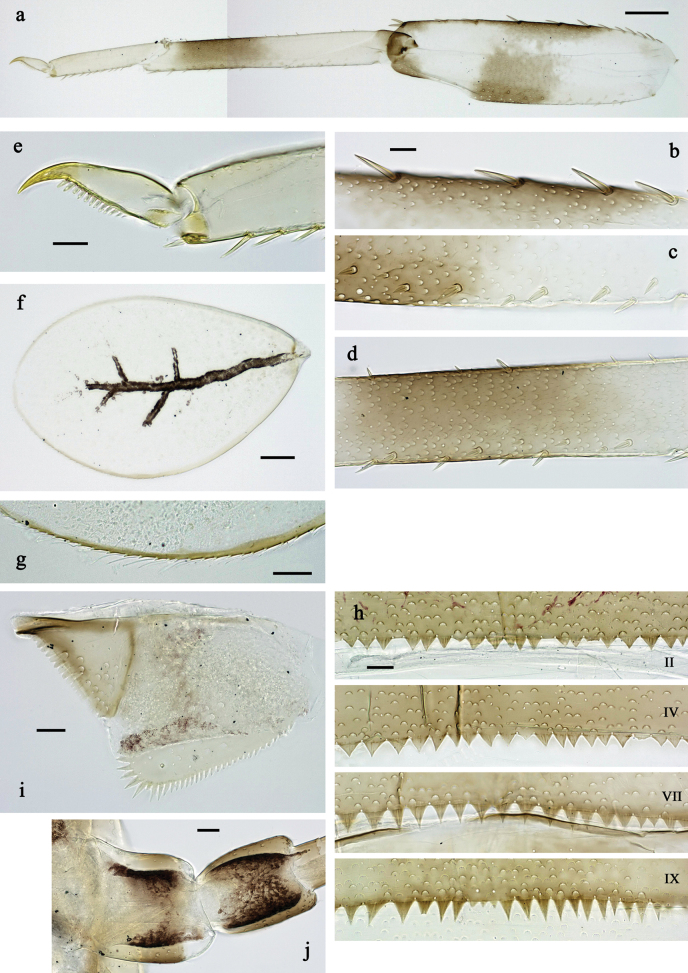
*Labiobaetis karen* sp. nov., larva morphology: a. Fore leg; b. Outer margin of fore femur; c. Inner margin of fore femur; d. Section of fore tibia; e. Fore claw; f, g. Tergalius III; h. Posterior margins of abdominal terga; i. Paraproct; j. Antennal base. Scale bars: 50 µm (a); 20 µm (f); 10 µm (b–e, g–j).

**Larva** (Figs 20–22). Body length 3.5–4.7 mm. Caudalii ~1/2 body length, paracercus ~0.4× body length. Antenna: ~2× as long as head length.

***Colouration*** (Fig. 20a–c). Head and thorax dorsally ochre, head and pronotum with dark brown markings, mesonotum with pronounced dark brown distolateral dots as in Fig. 20a; abdomen dorsally dark reddish-brown, paler in middle area, laterally pale brown, segment I ochre. Fore protoptera ochre. Head, thorax and abdomen ventrally mainly beige, abdominal segments laterally dark reddish brown. Legs pale brown, femur with dark brown, triangular marking at inner, distomedial margin, dark brown streak at outer margin, and dark brown marking at apex; tibia dark brown in subdistal area. Caudalii pale brown. Antennal scapus and pedicellus with dark brown hypodermal, lateral colouration.

***Antenna*** (Fig. 22j) with scape and pedicel sub cylindrical, distolateral process at scape absent.

***Labrum*** (Fig. 21a, b). Sub-rectangular, length 0.7× maximum width. Distal margin with medial emargination and small process. Dorsally with medium, fine, simple setae scattered over surface; submarginal arc of ~15 long, clavate setae on each side. Ventrally with marginal row of setae composed of anterolateral long, feathered setae and medial long, bifid setae.

***Right mandible*** (Fig. 21i). Incisor and kinetodontium fused. Incisor with five denticles; kinetodontium with three denticles, inner margin of innermost denticle with row of thin setae. Prostheca robust, apically denticulate. Margin between prostheca and mola almost straight. Tuft of setae on proximal corner of mola present.

***Left mandible*** (Fig. 21h). Incisor and kinetodontium fused. Incisor with five denticles, kinetodontium with four denticles. Prostheca robust, apicolaterally with small denticles and comb-shaped structure. Margin between prostheca and mola straight. Tuft of setae on proximal corner of mola absent.

Both mandibles with lateral margins almost straight.

***Hypopharynx and superlinguae*** (Fig. 21e). Lingua approx. as long as superlinguae. Lingua longer than broad, subdistally slightly expanded; medial tuft of stout setae well developed. Superlinguae with lateral margins rounded; fine, long, simple setae along distal margin.

***Maxilla*** (Fig. 21f, g). Galea-lacinia ventrally with two simple, apical setae below canines. Medially with one feathered, spine-like seta and four or five medium to long simple setae. Maxillary palp longer than length of galea-lacinia; 2-segmented; palp segment II slightly longer than segment I; setae on maxillary palp fine, simple, scattered over surface of segments I and II; apex of last segment with distolateral excavation, apically rounded.

***Labium*** (Fig. 21c, d). Glossa basally broad, narrowing toward apex; shorter than paraglossa; inner margin with ~7 robust, spine-like setae, distalmost seta longer; apex with two long and one medium, robust, apically pectinate setae; outer margin with ~3 spine-like setae; ventral surface with fine, simple, scattered setae. Paraglossa sub-rectangular, slightly curved inward; apex rounded; with three rows of long, robust, distally pectinate setae in apical area and three or four medium, simple setae in anteromedial area; dorsally with four long, spine-like setae near inner margin. Labial palp with segment I approx. as long as segments II and III combined. Segment II with rounded, thumb-like, distomedial protuberance; distomedial protuberance 0.7× width of base of segment III; dorsally with two spine-like setae near outer margin. Segment III subrectangular, inner distal margin slightly concave; length approx. as maximal width; ventrally covered with short, spine-like, simple setae and short, fine, simple setae.

***Hind protoptera*** absent.

***Legs*** (Fig. 22a–e). Ratio of foreleg segments 1.2:1.0:0.5:0.2, middle leg 1.1:1.0:0.4:0.2, hind leg 1.2:1.0:0.4:0.2. ***Femur***. Femur length ~3.5× maximum width. Outer margin with row of 10–12 spine-like setae; length of setae ~0.19× maximum width of femur. Apex rounded, with pair of spine-like setae and short, stout, apically blunt setae. Stout, lanceolate, pointed setae scattered along inner margin; femoral patch absent on fore and middle legs, rudimentary on hind leg. ***Tibia.*** Outer margin with row of short, stout, apically blunt setae, distalmost seta larger. Inner margin with two rows of medium spine-like setae; on apex tuft of fine, simple setae. Patella-tibial suture present on basal 1/3. ***Tarsus.*** Outer margin almost bare. Inner margin with row of curved, spine-like setae increasing in length distally. ***Claw*** with one row of 9–12 denticles; distally pointed.

***Abdominal terga*** (Fig. 22h). Surface with irregular rows of U-shaped scale bases and fine, simple, scattered setae. Posterior margin of terga: I smooth, without spines, II–IX with triangular, pointed spines, becoming longer and sharper toward end of abdomen.

***Abdominal sterna***. Posterior margin of sterna: I–VI smooth, without spines; VII–IX with triangular spines.

***Tergalii*** (Fig. 22f, g). Present on segments II–VII. Margin with small denticles intercalating fine, simple setae. Tracheae partly extending from main trunk to inner and outer margins. Tergalius IV somewhat longer than segments V; tergalius VII as long as segment VIII.

***Paraproct*** (Fig. 22i). Distally not expanded, with ~32 stout, marginal spines. Surface scattered with U-shaped scale bases and fine, simple setae. Cercotractor with numerous small, marginal spines.

##### Imago.

Unknown.

##### Etymology.

The species is dedicated to the indigenous Karen people in Thailand.

##### Distribution.

Thailand (Fig. 32d).

#### ﻿ Labiobaetis septem sp. nov.

Taxon classificationAnimaliaEphemeropteraBaetidae

7F2B3EFB-EAE0-5C8F-B657-3971EAE67ECB

https://zoobank.org/FE88795D-E801-4792-A7DC-E149DE1FB383

**Figure 23.**
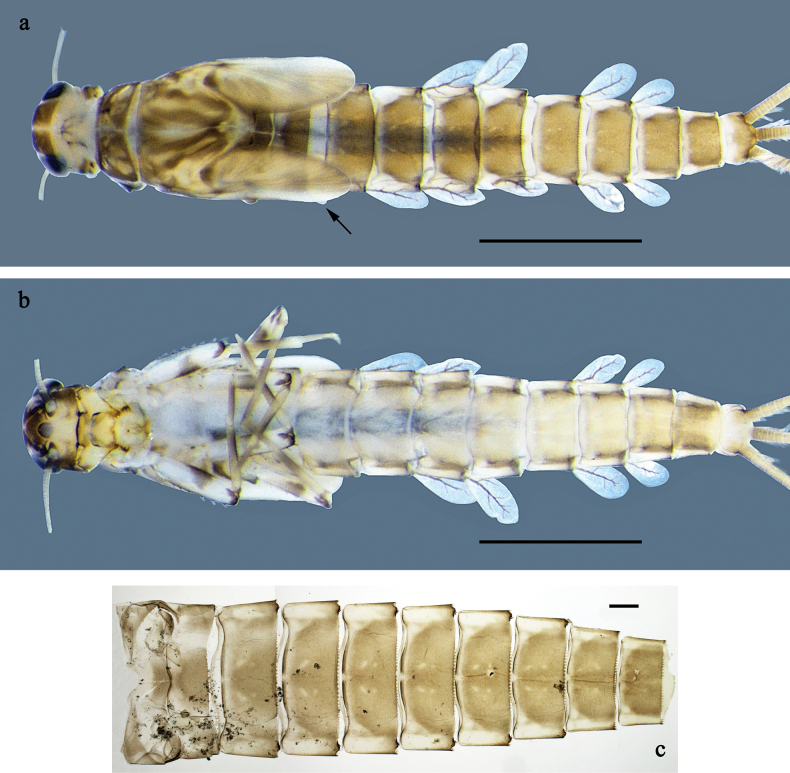
*Labiobaetis septem* sp. nov., larva (morphotype A): a. Habitus, dorsal view; b. Habitus, ventral view; c. Abdomen, dorsal view. Scale bars: 1 mm (a, b); 100 µm (c).

**Figure 24.**
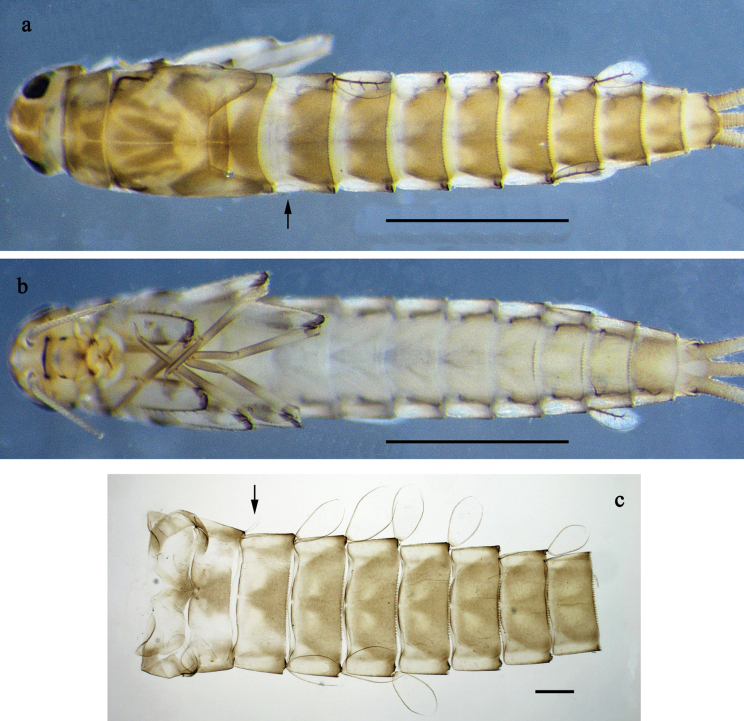
*Labiobaetis septem* sp. nov., larva (morphotype B): a. Habitus, dorsal view; b. Habitus, ventral view; c. Abdomen, dorsal view. Scale bars: 1 mm (a, b); 100 µm (c).

**Figure 25.**
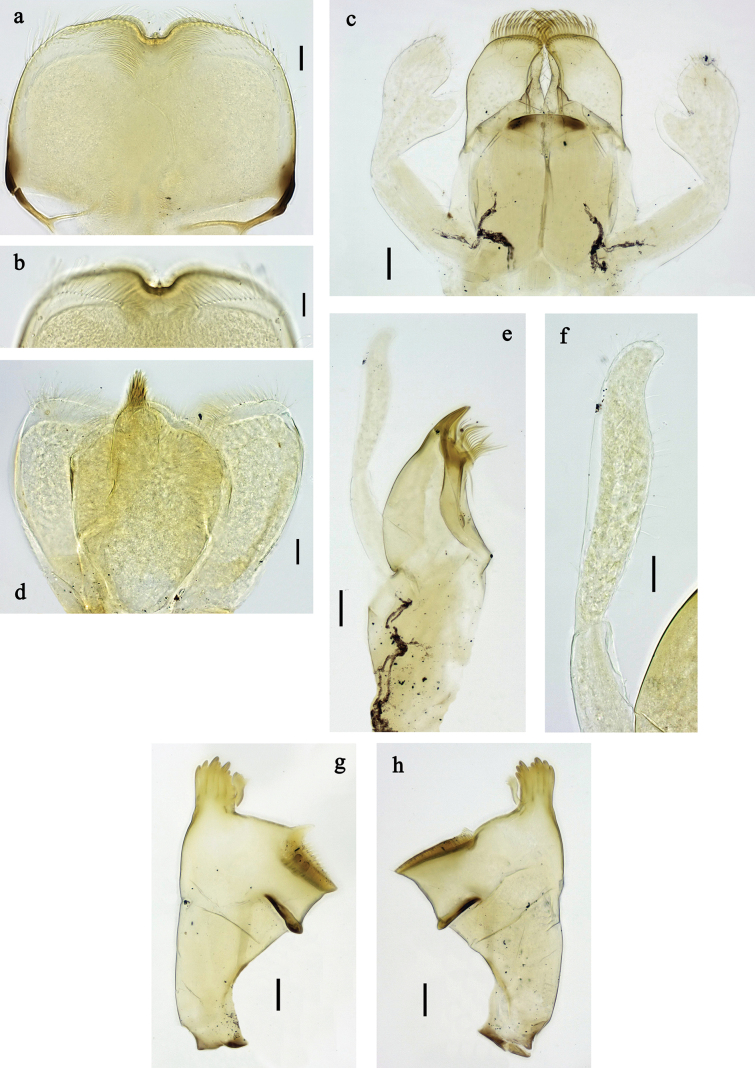
*Labiobaetis septem* sp. nov., larva morphology: a. Labrum; b. Section of labrum, dorsal focus; c. Labium; d. Hypopharynx and superlinguae; e. Maxilla; f. Maxillary palp; g. Left mandible; h. Right mandible. Scale bars: 20 µm (c, e, g, h); 10 µm (a, b, d, f).

**Figure 26.**
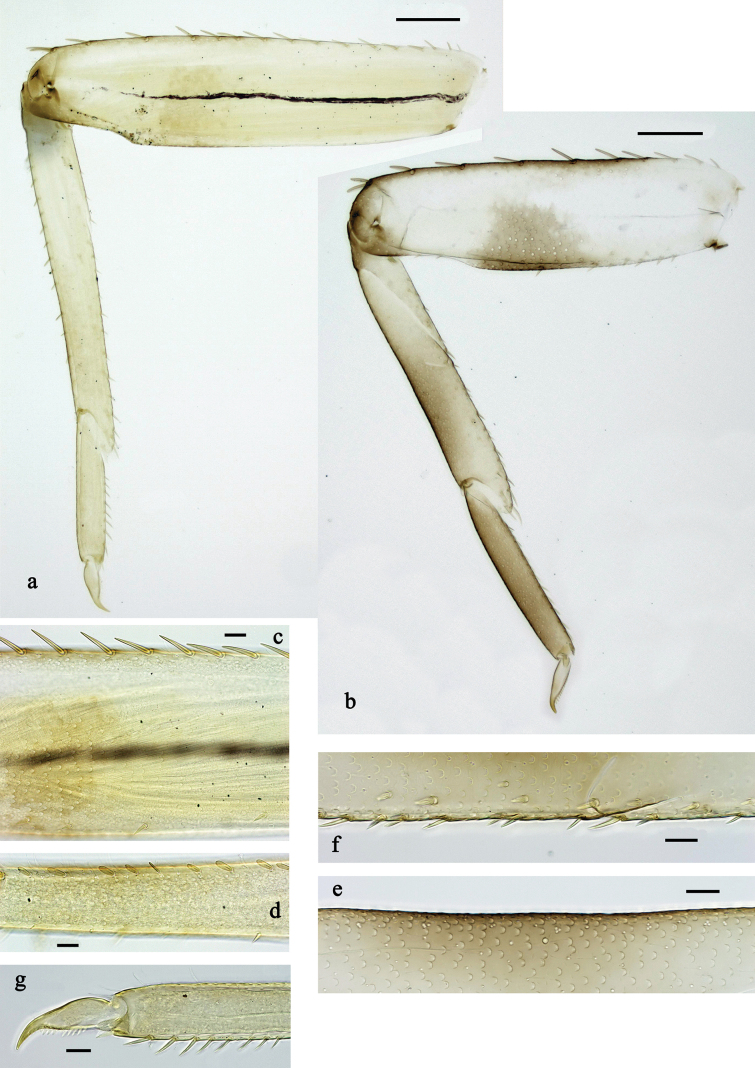
*Labiobaetis septem* sp. nov., larva morphology: a. Fore leg (morphotype A); b. Fore leg (morphotype B); c. Section of fore femur (morphotype A); d. Section of fore tibia (morphotype A); e. Outer margin of tibia (morphotype B); f. Inner margin of fore tibia (morphotype B); g. Fore tarsus and claw. Scale bars: 50 µm (a, b); 10 µm (c–g).

**Figure 27.**
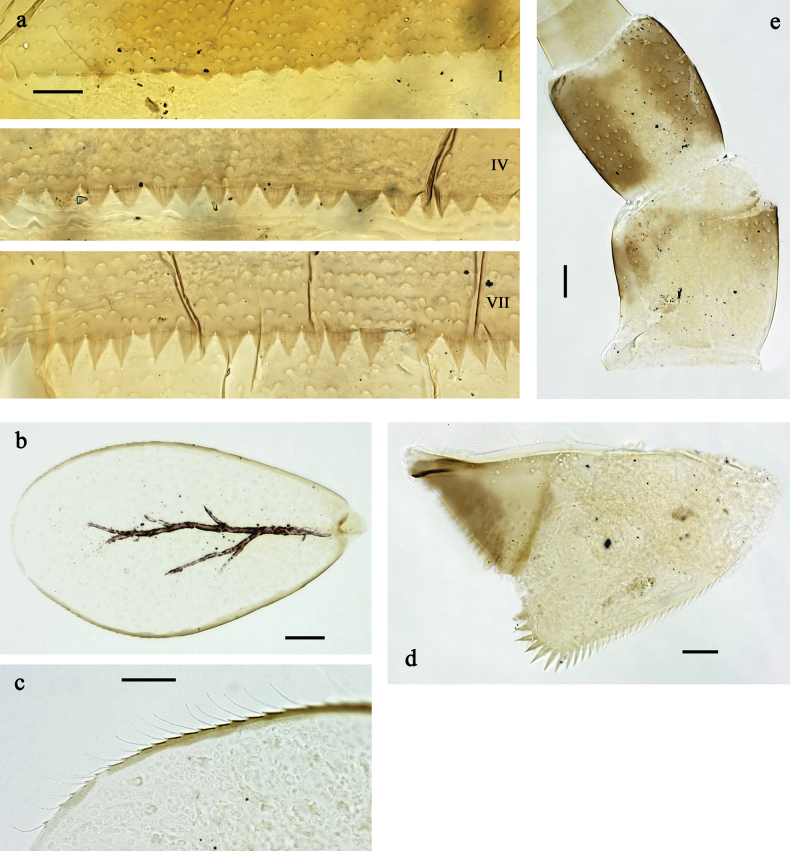
*Labiobaetis septem* sp. nov., larva morphology: a. Posterior margins of abdominal terga; b, c. Tergalius III; d. Paraproct; e. Antennal base. Scale bars: 20 µm (b); 10 µm (a, c–e).

Figs 23, 24, 25, 26, 27

##### Type material.

***Holotype*.** Thailand • larva; Tak Prov., Mueang Distr., Klong Lan Sang; 16°46'53"N, 99°01'16"E; 253 m; 25.xii.2017; leg. C. Suttinun; on slide; GBIFCH01223075; VMCMU. ***Paratypes*.** 18 larvae; same data as holotype; 2 on slides; GBIFCH01223076, GBIFCH01221815 (legs); MZL; 16 in alcohol; GBIFCH00829292; GBIFCH00763850; MZL; GBIFCH00763838; VMCMU • 5 larvae; Kanchanaburi Prov., Thong Pha Phum Distr., Pra Chum Mai; 14°34'58"N, 98°34'52"E; 269 m; 25.v.2017; leg. C. Suttinun; 1 on slide; GBIFCH00596155; MZL; 4 in alcohol; GBIFCH00763825; VMCMU • 1 larva; Loei Prov., Wang Saphung Distr., Nam Thob; 17°15'37"N, 101°34'53"E; 376 m; 17.xii.2018; leg. C. Suttinun; on slide; GBIFCH01223086; VMCMU • 4 larvae; Loei Prov., Wang Saphung Distr., Ban Nam Thob; 17°16'11"N, 101°35'51"E; 318 m; 17.xii.2018; leg. C. Suttinun; 1 on slide; GBIFCH00829294 (legs, abdomen); MZL; 3 in alcohol; GBIFCH00763837; MZL • 1 larva; Chiang Mai Prov., Mae On Distr., Huai Teen Tok; 18°52'03"N, 99°19'19"E; 760 m; 21.xi.2018; leg. C. Suttinun; on slide; GBIFCH00607173; VMCMU • 1 larva; Chiang Mai Prov., Chiang Dao Distr., Huai Mae Mae; 19°19'19"N, 98°52'51"E; 809 m; 20.xi.2018; leg. C. Suttinun; on slide; GBIFCH01221823; MZL.

##### Other material.

Thailand • 11 larvae; Tak Prov., Mueang Distr., Klong Lan Sang; 16°46'53"N, 99°01'16"E; 253 m; 25.xii.2017; leg. C. Suttinun; 3 on slides; GBIFCH00607174; VMCMU; GBIFCH00975857, GBIFCH00975858; MZL; 8 in alcohol; GBIFCH00763818, GBIFCH00975856, GBIFCH00763830; VMCMU • 5 larvae; Tak Prov., Mueang Distr., Tha Le; 16°44'28"N, 99°02'43"E; 327 m; 26.xii.2017; leg. C. Suttinun; 1 on slide; GBIFCH01221817; MZL; 4 in alcohol; GBIFCH00980863; MZL; GBIFCH00763841; VMCMU • 4 larvae; Chang Mai Prov., Mae On Distr., Huai Teen Tok; 18°52'03''N, 99°19'19''E; 760 m; 17.xi.2023; leg. C. Suttinun; VMCMU.

##### Diagnosis.

**Larva.** Following combination of characters differentiate *L. septem* sp. nov. from other species of the group *sumigarensis*: tergalii present on abdominal segments I–VII; abdomen dorsally uniform brown, or brown with crown-like pattern; femur with 9–15 spine-like setae at outer margin; tibia with row of short, apically rounded setae at outer margin, or bare with one medium, apically rounded seta distally; claw with 10–14 denticles.

##### Description.

**Larva** (Figs 23–26). Body length 3.6–4.6 mm. Cerci ~1/2 body length, paracercus ~1/3 body length. Antenna: ~2× as long as head length.

***Colouration*** (Figs 23a–c, 24a–c). Head and thorax dorsally yellow-brown with dark grey pattern as in Fig. 23a; abdomen dorsally rather uniform brown, or brown with crown-like pattern. Fore protoptera yellow-brown to brown. Head, thorax and abdomen ventrally mainly pale grey to yellowish, abdominal segments laterally darker, and off-white along margins, abdominal segment IX darker. Legs with femur off-white to grey, with triangular, dark grey, distomedial marking at inner margin and dark grey apex; tibia yellow-brown in basal 1/2 and grey in distal 1/2; tarsus grey. Caudalii grey-brown, with dark brown distomedial section. Antennal scapus and pedicellus laterally dark grey-brown.

***Antenna*** (Fig. 26e) with scape and pedicel sub cylindrical, distolateral process at scape absent.

***Labrum*** (Fig. 25a, b). Sub-rectangular, length 0.7× maximum width. Distal margin with medial emargination and small process. Dorsally with medium, fine, simple setae scattered over surface; submarginal arc of ~17 long, clavate setae on each side. Ventrally with marginal row of setae composed of anterolateral long, feathered setae and medial long, bifid setae.

***Right mandible*** (Fig. 25h). Incisor and kinetodontium fused. Incisor with five denticles; kinetodontium with three denticles, inner margin of innermost denticle with row of thin setae. Prostheca robust, apically denticulate. Margin between prostheca and mola almost straight, with few minute denticles. Tuft of setae on proximal corner of mola present.

***Left mandible*** (Fig. 25g). Incisor and kinetodontium fused. Incisor with five denticles, kinetodontium with three denticles. Prostheca robust, apicolaterally with small denticles and comb-shaped structure. Margin between prostheca and mola straight, with minute denticles towards subtriangular process. Tuft of setae on proximal corner of mola absent.

Both mandibles with lateral margins almost straight.

***Hypopharynx and superlinguae*** (Fig. 25d). Lingua approx. as long as superlinguae, longer than broad, subdistally slightly expanded; medial tuft of stout setae well developed. Superlinguae with lateral margins rounded; fine, long, simple setae along distal margin.

***Maxilla*** (Fig. 25e, f). Galea-lacinia ventrally with two simple, apical setae below canines. Medially with one feathered, spine-like seta three medium to long, simple setae. Maxillary palp longer than length of galea-lacinia; 2-segmented; palp segment II 1.3× length of segment I; setae on maxillary palp fine, simple, scattered over surface of segments I and II; apex of last segment with well-developed distolateral excavation, apically rounded.

***Labium*** (Fig. 25c). Glossa basally broad, narrowing toward apex; shorter than paraglossa; inner margin with ~5 spine-like setae; apex with two long and one medium, robust, apically pectinate setae; outer margin with ~5 spine-like setae; ventral surface with fine, simple, scattered setae. Paraglossa sub-rectangular, slightly curved inward; apex rounded; with three rows of long, robust, distally pectinate setae in apical area and three medium, simple setae in anteromedial area; dorsally with three long, spine-like setae near inner margin. Labial palp with segment I 0.8× length of segments II and III combined. Segment II with rounded, thumb-like, distomedial protuberance; distomedial protuberance 0.8× width of base of segment III; dorsally with two or three spine-like setae near outer margin. Segment III subrectangular; length ~1.2× maximal width; ventrally covered with short, spine-like, simple setae and short, fine, simple setae.

***Hind protoptera*** absent.

***Legs*** (Fig. 26a–g). Ratio of foreleg segments 1.2:1.0:0.4–0.6:0.2, middle leg 1.1:1.0:0.4:0.1, hind leg 1.2:1.0:0.5–0.6:0.2. ***Femur***. Femur length ~4× maximum width. Outer margin with row of 9–15 spine-like setae; length of setae ~0.24× maximum width of femur. Apex rounded, with pair of spine-like setae and some short, apically blunt setae. Stout, lanceolate, pointed setae scattered along inner margin; femoral patch rudimentary on foreleg, absent on middle and hind leg. ***Tibia.*** Outer margin with row of short, stout, apically blunt setae, distalmost seta larger, or bare with one stout, apically rounded seta distally. Inner margin with two rows of medium spine-like setae; on apex tuft of fine, simple setae. Patella-tibial suture present on basal 1/3. ***Tarsus.*** Outer margin almost bare. Inner margin with row of curved, spine-like setae. ***Claw*** with one row of 10–14 denticles; distally pointed.

***Abdominal terga*** (Fig. 27a). Surface with irregular rows of U-shaped scale bases and fine, simple, scattered setae. Posterior margin of terga: I–IX with triangular spines, I wide and short, II–IX becoming longer and sharper toward end of abdomen.

***Abdominal sterna***. Posterior margin of sterna: I–VI smooth, without spines; VII–IX with triangular spines.

***Tergalii*** (Fig. 27b, c). Present on segments I–VII. Margin with small denticles intercalating fine, simple setae. Tracheae partly extending from main trunk to inner and outer margins. Tergalius I small, ~1/3 length of segment II; tergalius IV somewhat longer than segment V; tergalius VII as long as segment VIII.

***Paraproct*** (Fig. 27d). Distally not expanded, with ~29 stout, marginal spines. Surface scattered with U-shaped scale bases and fine, simple setae. Cercotractor with numerous small, marginal spines.

##### Remark.

The species appears with two different morphotypes, which partly share the same locations: based on our material, morphotype A is characterised by a dorsally rather uniform brown abdomen, a row of setae at outer margin of tibia, 10–15 setae at outer margin of femur, and a claw with 10 or 11 denticles; morphotype B has a crown-like pattern dorsally on the abdomen, outer margin of tibia almost bare, femur with nine or ten setae at outer margin, and claw with 12–14 denticles (see in discussion section). Holotype and paratypes correspond with morphotype A, specimens with morphotype B are mentioned as other material.

##### Imago.

Unknown.

##### Etymology.

The Latin word *septem*, meaning seven, refers to the seven pairs of tergalii of this species. All other known species of the group *sumigarensis* have only six pairs of tergalii.

##### Distribution.

Thailand (Fig. 32d).

#### ﻿ Labiobaetis ranongensis sp. nov.

Taxon classificationAnimaliaEphemeropteraBaetidae

3D0C663C-3DC6-5DD2-8BFF-78E82C070AD7

https://zoobank.org/B38C906E-6CFD-4A16-B283-26BAB4798FCD

Figs 28, 29, 30

##### Type material.

***Holotype*.** Thailand • larva; Ranong Prov., Mueang Distr., Huai Nam Sai; 09°43'25"N, 98°36'29"E; 50 m; 20.vi.2018; leg. C. Suttinun; on slide; GBIFCH00980864; VMCMU. ***Paratypes*.** Thailand • 4 larvae; Ranong Prov., Mueang Distr., Punyaban Waterfall; 10°03'54"N, 98°40'13"E; 52 m; 19.vi.2018; leg. C. Suttinun; 1 on slide; GBIFCH01223077; MZL; 3 in alcohol; GBIFCH00763828; VMCMU • 14 larvae; Ranong Prov., Mueang Distr., Huai Por Ta Hin Rao; 09°52'08"N, 98°37'32"E; 20 m; 20.vi.2018; leg. C. Suttinun; 1 on slide; GBIFCH00980860; MZL; 13 in alcohol; GBIFCH00763826, GBIFCH00763834; VMCMU; GBIFCH00975850; MZL • 1 larva; Ranong Prov., Mueang Distr., Klong Nok Ngang; 09°42'22"N, 98°34'39"E; 11 m; 21.vi.2018; leg. C. Suttinun; on slide; GBIFCH00975861; MZL • 5 larvae; Ranong Prov., Mueang Distr., Huai Por Ta; 10°02'03"N, 98°39'29"E; 22 m; 19.iv.2018; leg. C. Suttinun; in alcohol; GBIFCH00763835; VMCMU.

##### Diagnosis.

**Larva.** Following combination of characters differentiate *L. ranongensis* sp. nov. from other species of the group *sumigarensis*: abdomen dorsally yellow-brown, basal parts of terga paler, posterior parts with a darker, slightly crown-like marking; labial palp segment III subrectangular, segment II with thumb-like protuberance with straight distal margin and rounded lateral margin; maxillary palp longer than galea-lacinia, terminal segment with well-developed, distolateral excavation; left mandible with margin between prostheca and mola straight, with minute marginal and submarginal denticles; abdominal tergites II–IX with triangular, sharply pointed spines on posterior margins; paraproct distally not expanded, with ~28 spines.

##### Description.

**Figure 28.**
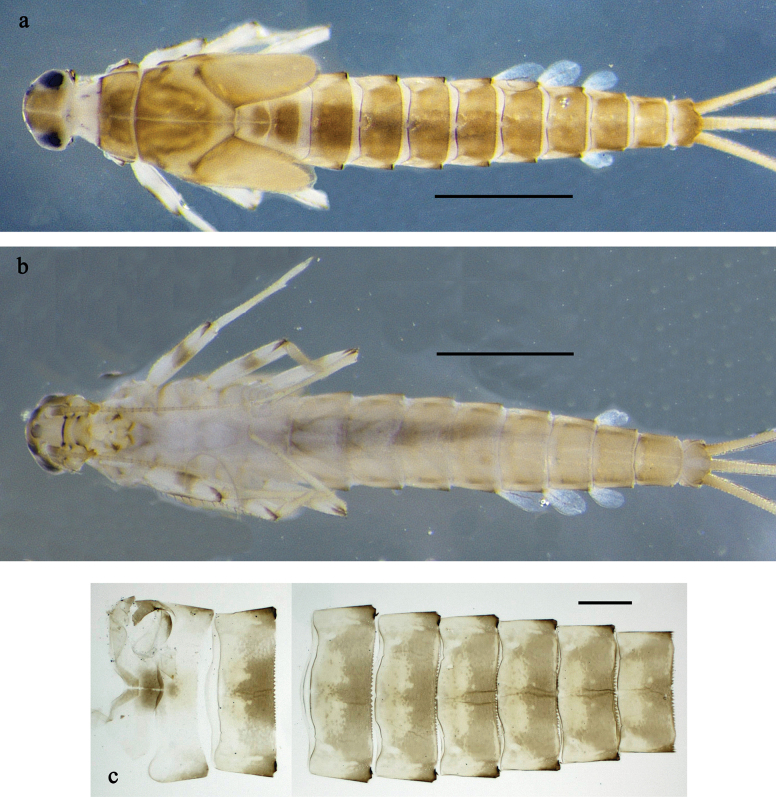
*Labiobaetis ranongensis* sp. nov., larva: a. Habitus, dorsal view; b. Habitus, ventral view; c. Abdomen, dorsal view. Scale bars: 1 mm (a, b); 100 µm (c).

**Figure 29.**
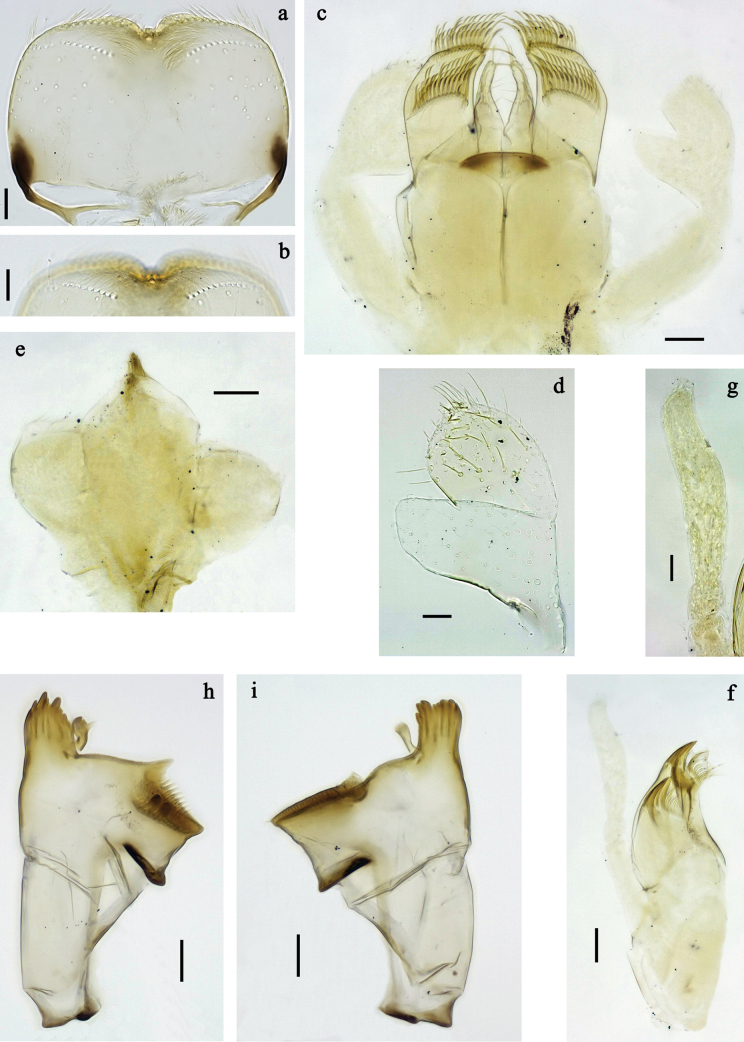
*Labiobaetis ranongensis* sp. nov., larva morphology: a. Labrum; b. Section of labrum, dorsal focus; c. Labium; d. Labial palp; e. Hypopharynx and superlinguae; f. Maxilla; g. Maxillary palp; h. Left mandible; i. Right mandible. Scale bars: 20 µm (c, e, f, h, i); 10 µm (a, b, d, g).

**Figure 30.**
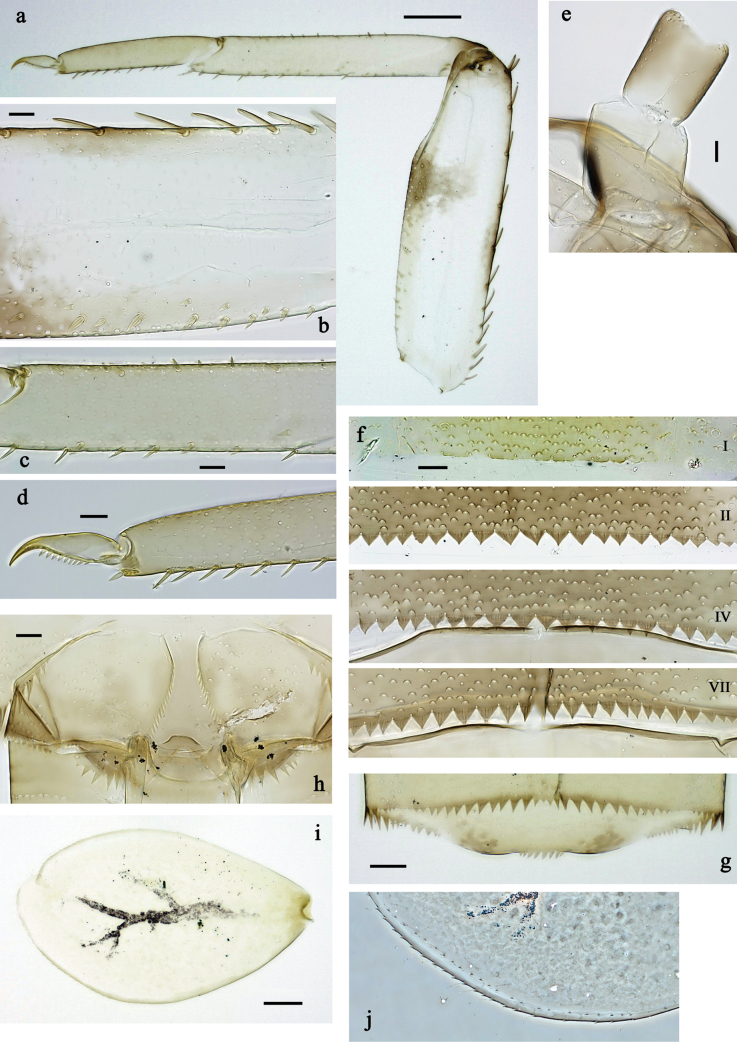
*Labiobaetis ranongensis* sp. nov., larva morphology: a. Hind leg; b. Section of hind femur; c. Section of hind tibia; d. Hind tarsus and claw; e. Antennal base; f. Posterior margins of abdominal terga; g. Posterior margins of abdominal segment IX; h. Paraprocts; i, j. Tergalius V. Scale bars: 50 µm (a); 20 µm (g); 10 µm (b–f, h–j).

**Larva** (Figs 28–30). Body length 3.3–4.9 mm. Cerci ~1/2 body length, paracercus ~0.4× body length. Antenna: ~2× as long as head length.

***Colouration*** (Fig. 28a–c). Head and thorax dorsally yellow-brown, with dark brown markings; abdomen dorsally yellow-brown, anterior parts of abdominal segments paler, posterior parts with a darker, slightly crown-like marking; Fore protoptera yellow-brown. Thorax ventrally off-white; abdomen ventrally pale grey-brown. Legs with femur off-white, with distomedial, triangular grey-brown marking, apex grey-brown; tibia and tarsus yellow-brown. Caudalii yellow-brown.

***Antenna*** (Fig. 30e) with scape and pedicel sub cylindrical, distolateral process at scape absent.

***Labrum*** (Fig. 29a, b). Sub-rectangular, length 0.7× maximum width. Distal margin with medial emargination and small process. Dorsally with medium, fine, simple setae scattered over surface; submarginal arc of ~18 long, clavate setae on each side. Ventrally with marginal row of setae composed of anterolateral long, feathered setae and medial long, bifid setae.

***Right mandible*** (Fig. 29i). Incisor and kinetodontium fused. Incisor with five denticles; kinetodontium with three denticles, inner margin of innermost denticle with row of thin setae. Prostheca robust, apically denticulate. Margin between prostheca and mola slightly convex, with minute denticles. Tuft of setae on proximal corner of mola present.

***Left mandible*** (Fig. 29h). Incisor and kinetodontium fused. Incisor with five denticles, kinetodontium with three denticles. Prostheca robust, apicolaterally with small denticles and comb-shaped structure. Margin between prostheca and mola straight, with marginal and submarginal, minute denticles. Tuft of setae on proximal corner of mola absent.

Both mandibles with lateral margins almost straight.

***Hypopharynx and superlinguae*** (Fig. 29e). Lingua longer than superlinguae, longer than broad, subdistally slightly expanded; medial tuft of stout setae well developed. Superlinguae with lateral margins rounded; fine, long, simple setae along distal margin.

***Maxilla*** (Fig. 29f, g). Galea-lacinia ventrally with two simple, apical setae below canines. Medially with one feathered, spine-like seta and three medium to long, simple setae. Maxillary palp longer than length of galea-lacinia; 2-segmented; palp segment II ~1.2× length of segment I; setae on maxillary palp fine, simple, scattered over surface of segments I and II; apex of last segment with well-developed distolateral excavation, apically rounded.

***Labium*** (Fig. 29c, d). Glossa basally broad, narrowing toward apex; shorter than paraglossa; inner margin with ~7 robust, spine-like setae, increasing in length distally; apex with two long and one medium, robust, apically pectinate setae; outer margin with ~4 spine-like setae; ventral surface with fine, simple, scattered setae. Paraglossa sub-rectangular, slightly curved inward; apex rounded; with three rows of long, robust, distally pectinate setae in apical area and four medium, simple setae in anteromedial area; dorsally with three or four long, spine-like setae near inner margin. Labial palp with segment I approx. as long as segments II and III combined. Segment II with thumb-like protuberance with straight distal margin and rounded lateral margin; distomedial protuberance ~0.9× width of base of segment III; dorsally with two spine-like setae near outer margin. Segment III subrectangular; length approx. as maximal width; ventrally covered with short, spine-like, simple setae and short, fine, simple setae. Mentum distally with dark grey-brown marking.

***Hind protoptera*** absent.

***Legs*** (Fig. 30a–d). Ratio of foreleg segments 1.1:1.0:0.4:0.1, middle leg 1.1:1.0:0.4:0.1, hind leg 1.2:1.0:0.4:0.1. ***Femur***. Femur length ~3.7× maximum width. Outer margin with row of 9–15 spine-like setae; length of setae ~0.30× maximum width of femur. Apex rounded, with pair of spine-like setae and usually few short, apically blunt setae. Stout, lanceolate, pointed setae scattered along inner margin; femoral patch absent on fore and middle legs, rudimentary on hind leg. ***Tibia.*** Outer margin with row of short, apically blunt setae, distalmost seta larger. Inner margin with two rows of medium spine-like setae; on apex tuft of fine, simple setae. Patella-tibial suture present on basal 1/3. ***Tarsus.*** Outer margin almost bare. Inner margin with row of curved, spine-like setae. ***Claw*** with one row of 7–14 denticles; distally pointed.

***Abdominal terga*** (Fig. 30f, g). Surface with irregular rows of U-shaped scale bases and fine, simple, scattered setae. Posterior margin of terga: I smooth, without spines, II–IX with triangular, sharply pointed spines, becoming longer, narrower and sharper toward end of abdomen.

***Abdominal sterna*** (Fig. 30g). Posterior margin of sterna: I–VI smooth, without spines; VII–IX with triangular spines, spines on segment IX not continuous.

***Tergalii*** (Fig. 30i, j). Present on segments II–VII. Margin with small denticles intercalating fine, simple setae. Tracheae partly extending from main trunk to inner and outer margins. Tergalius IV somewhat longer than segment V; tergalius VII as long as 3/4 length of segment VIII.

***Paraproct*** (Fig. 30h). Distally not expanded, with ~30 stout, marginal spines. Surface scattered with U-shaped scale bases and fine, simple setae. Cercotractor with numerous small, marginal spines.

##### Imago.

Unknown.

##### Etymology.

The species name refers to Ranong Prov., where the type locality is located.

##### Distribution.

Thailand (Fig. 32d).

### ﻿Key to the *Labiobaetis* species of continental Southeast Asia (larvae)

**Table:** 

1	Labrum dorsally with arc of simple setae	**2**
–	Labrum dorsally with arc of feathered or clavate setae	**8**
2(1)	With well-developed hind protoptera (*batakorum* species group)	**3**
–	With minute hind protoptera (*numeratus* species group)	**5**
3(2)	Abdomen dorsally rather uniform brown to dark brown, paler in middle area, tergum V not much brighter (Fig. 1a)	***L. mon* sp. nov.**
–	Abdomen dorsally with lively pattern, tergum V much brighter than neighbouring terga (Figs 1d, 4a)	**4**
4(3)	Abdomen dorsally dark grey with yellowish pattern as in Fig. 4a, especially yellowish oval markings on terga III and V	***L. lahu* sp. nov.**
–	Abdomen dorsally grey-brown, laterally whitish with black markings, terga V and X brighter (Fig. 1d)	***L. multus***
5(2)	Left mandible with angular hump at margin between prostheca and mola (Fig. 11h)	***L. angularis* sp. nov.**
–	Left mandible without angular hump at margin between prostheca and mola	**6**
6(5)	Abdomen dorsally grey-brown, tergum II laterally darker with dark brown, roundish, distomedial marking, tergum V dark brown	***L. tonsator* sp. nov.**
–	Abdomen dorsally rather uniform brown	**7**
7(6)	Labial palp segment III approx. as long as wide at base (Müller-Liebenau 1984: fig. 11g); claw with 10–12 denticles; paraproct with patch of notched scales (similar to Kaltenbach and Gattolliat 2019a: fig. 3g, h)	***L. numeratus***
–	Labial palp segment III rather short (length 0.8× width at base; Fig. 8d); claw with ~14 denticles; paraproct without patch of notched scales	***L. tenasserimensis* sp. nov.**
8(1)	Labrum dorsally with arc of feathered setae (*operosus* species group)	**9**
–	Labrum dorsally with arc of clavate setae (*sumigarensis* species group)	**12**
9(8)	Labial palp segment II with broad thumb-like (lobed), distolateral protuberance, segment III oblong; maxillary palp segment II with slightly developed distolateral excavation (Kaltenbach and Gattolliat 2019b: fig. 36g, h)	**L. cf. paraoperosus**
–	Labial palp segment II with rather narrow or elongate thumb-like, distomedial protuberance (Figs 16a, 17c; Kaltenbach et al. 2022a: fig. 1i), segment III slightly pentagonal; maxillary palp segment II with well-developed, distolateral excavation (Fig. 17g)	**10**
10(9)	Labial palp segment II with thumb-like, distomedial protuberance, slightly bent distad; paraproct at inner, proximal margin with additional submarginal row of minute spines (Kaltenbach et al. 2022a: figs 1i, 2 f, g)	***L. brao***
–	Labial palp segment II with thumb-like, distomedial protuberance, directed laterally, proximal margin of protuberance slightly concave (Figs 16e, 17c); paraproct without additional submarginal row of minute spines	**11**
11(10)	Distomedial protuberance of labial palp segment II shorter than base of segment III (~0.8×) (Fig. 16e)	***L. operosus***
–	Distomedial protuberance of labial palp segment II elongated, longer than base of segment III (~1.2×) (Fig. 17c)	***L. nisaratae* sp. nov.**
12(8)	Tergalii on abdominal segments I–VII	***L. septem* sp. nov.**
–	Tergalii on abdominal segments II–VII	**13**
13(12)	Antennal scape with reduced, distolateral process (Müller-Liebenau 1984: fig. 6f)	***L. diffundus***
–	Antennal scape without distolateral process	**14**
14(13)	Thorax dorsally ochre with pronounced, dark brown, distolateral markings on mesonotum; abdomen dorsally dark reddish-brown, slightly paler in middle area, tergum I ochre; abdomen ventrally mainly beige, laterally dark reddish-brown (Fig. 20a, b)	***L. karen* sp. nov.**
–	Thorax dorsally pale brown, no pronounced distolateral markings on mesonotum; abdomen dorsally pale brown, ventrally rather uniform beige	**15**
15(14)	Posterior margin of abdominal terga with triangular spines, sometimes spaced; abdominal terga dorsally rather uniform brown (Kaltenbach et al. 2022a: figs 4d, 5c)	***L. kui***
–	Posterior margin of abdominal terga with triangular, sharply pointed spines (Fig. 31g); abdominal terga dorsally brown with crown-like pattern (paler in anterior parts of seg; Fig. 28c)	***L. ranongensis* sp. nov.**

**Figure 31.**
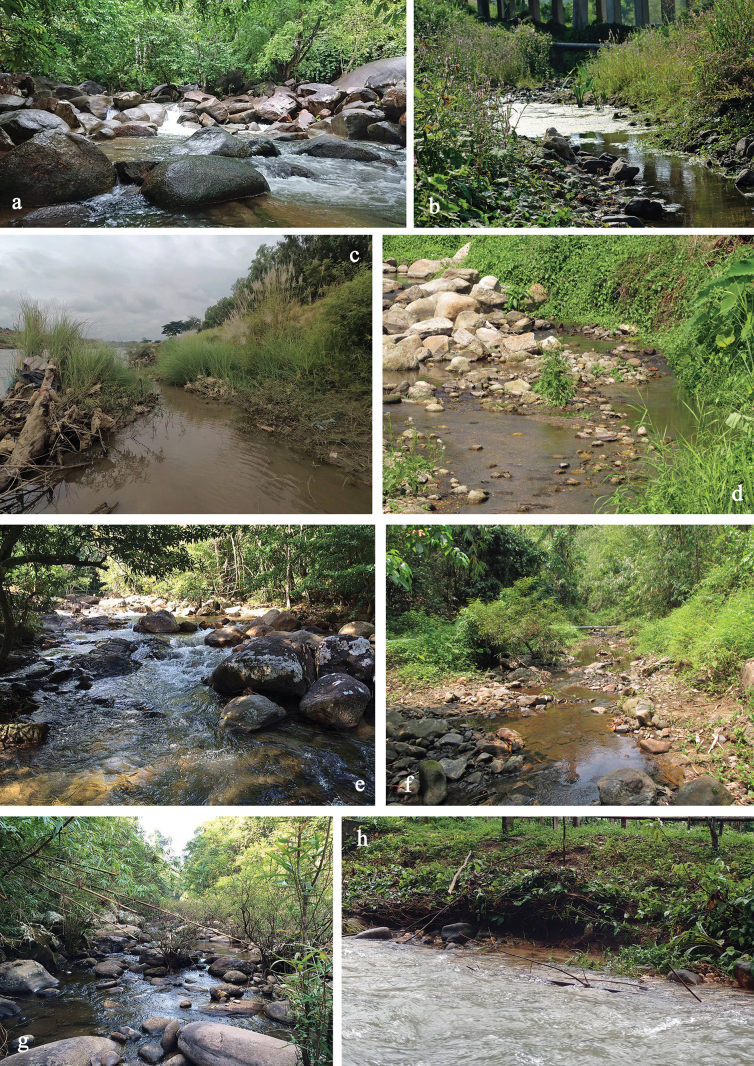
Habitats: a. *Labiobaetis multus*; b. *Labiobaetis mon* sp. nov. (TL) and *angularis* sp. nov. (TL); c. *Labiobaetis lahu* sp. nov.; d. *Labiobaetis tenasserimensis* sp. nov. (TL); e. *Labiobaetis tonsator* sp. nov. (TL); f. *Labiobaetis nisaratae* sp. nov. and *L. karen* sp. nov. (TL); g. *Labiobaetis septem* sp. nov.; h. *Labiobaetis ranongensis* sp. nov. (TL). TL: type locality.

**Figure 32.**
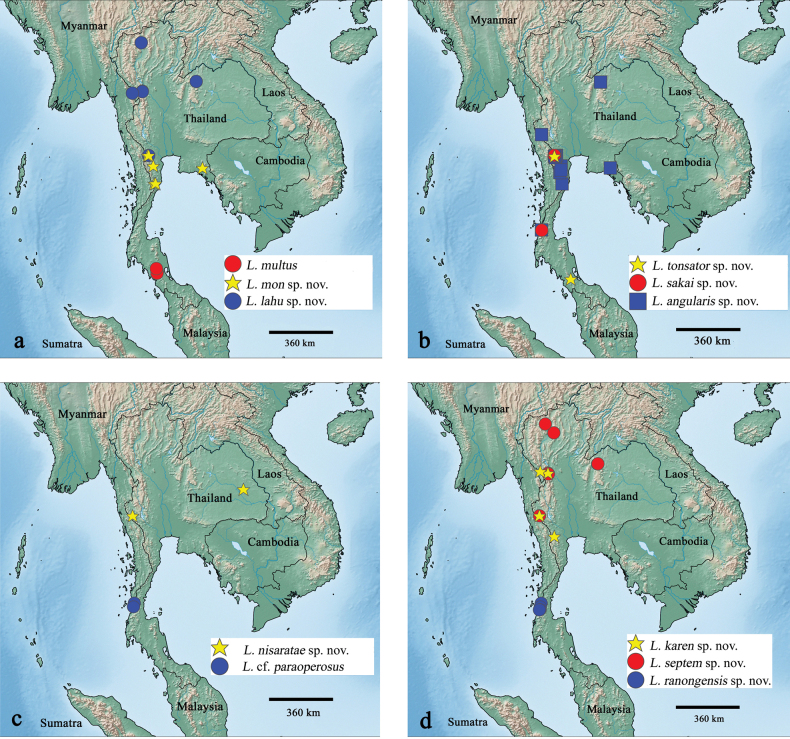
Distribution maps: a. *Labiobaetis batakorum* species group; b. *Labiobaetis numeratus* species group; c. *Labiobaetis operosus* species group; d. *Labiobaetis sumigarensis* species group.

## ﻿Discussion

### ﻿Assignment to *Labiobaetis*

For the assignment of the new species to *Labiobaetis* we refer to Kluge and Novikova (2014), Müller-Liebenau (1984), and McCafferty and Waltz (1995). *Labiobaetis* is defined by a number of characters (Kluge and Novikova 2014): antennal scape sometimes with a distolateral process (Fig. 18g); maxillary palp two segmented with excavation at inner distolateral margin of segment II, excavation may be poorly developed or absent (Figs 17g, 25f)); labium with paraglossae widened and glossae diminished; labial palp segment II with distomedial protuberance (Figs 5d, 14d, 17d). All these characters vary and may be secondarily lost (Kluge and Novikova 2014). The concept of *Labiobaetis* is also based on additional characters, summarised and discussed in Kaltenbach and Gattolliat (2018, 2019b).

The morphological species groups within *Labiobaetis* are primarily a working tool, but could also serve as a basis for future studies on the generic or subgeneric delimitation and phylogeny of this genus. Further investigation including the analysis of nuclear gene sequences may prove that some are natural groups.

### ﻿Genetics

We obtained COI sequences from all *Labiobaetis* species reported from Thailand, including the nine newly described species (Table 1). The interspecific genetic distances (COI; Kimura-2 parameter (K2P); Suppl. material 1) between all species strongly support the morphological differences and their species status. The minimum K2P distance between species of 10% (distance between *L. multus* and *L. mon* sp. nov.) is far beyond the generally accepted threshold of 3% divergence for mayflies (e.g., Ball et al. 2005; Kjærstad et al. 2012; Gattolliat et al. 2015). Furthermore, we estimated the genetic distances between the species from Thailand and all other species from Southeast Asia, as far as COI sequences were available (see Kaltenbach and Gattolliat 2019b, 2020; Kaltenbach et al. 2020a, 2022a). As result, the species status of all new species described in this study is supported.

The genetic distances between *Labiobaetis* species in Thailand (10–25%, K2P) is rather high, which is in line with the interspecific genetic distances found in Indonesia (11–24%; Kaltenbach and Gattolliat 2019b), Borneo (19–25%; Kaltenbach and Gattolliat 2020), the Philippines (15–27%; Kaltenbach et al. 2020a), and Cambodia (20–21%; Kaltenbach et al. 2022a). Ball et al. (2005) reported a mean interspecific, congeneric distance of 18% for mayflies from the United States and Canada. The intraspecific genetic distances in Thailand are very low as expected, always ranging from 0% to 3% (Suppl. material 1).

### The case of *Labiobaetis septem* sp. nov.

Interestingly, the new species *Labiobaetis septem* sp. nov. presents two different morphotypes. Morphotype A has a dorsally rather uniform brown abdomen (Fig. 23a, c), the legs display a row of stout, apically rounded setae at outer margin of the tibia (Fig. 26a, d), 10–15 spine-like *setae* at outer margin of the femur, and a claw with 10 or 11 denticles in one row. Morphotype B is characterised by a crown-like pattern dorsally on the abdomen (Fig. 25a, c), and legs with an almost bare outer margin of the tibia (Fig. 26b, e), a femur with nine or ten spine-like setae at outer margin, and a claw with 12–14 denticles. Partly, both morphotypes occur at the same location. We obtained for each morphotype three COI sequences, which are all identical. We also found a specimen with a mix between both morphotypes, a crown-like pattern dorsally on the abdomen like morphotype B, and legs with a row of stout, apically rounded setae at outer margin of the tibia like morphotype A; the COI sequence of this specimen is identical with the ones of morphotype A and B. Based on these findings, our reasonable interpretation is that we have a substantial morphological variation in abdominal pattern and leg setation in the new species *Labiobaetis septem* sp. nov. However, we remain prudent and consider only specimens of morphotype A as holotype and paratypes. Specimens of morphotype B or a mixed morphotype are listed as “other material”. Furthermore, we investigated specimens from a different location than the locations of *Labiobaetis septem* sp. nov. Their morphology is in line with morphotype B of *L. septem* sp. nov., with a crown-like pattern of the abdomen, and a tibia with no or just a few setae at outer margin. The femur has 7–9 spine-like setae at outer margin, the claw has 13 or 14 denticles. We obtained three COI sequences from this population with a genetic distance (K2P) of 20% to the sequences of *L. septem* sp. nov. Considering the morphological variability of *L. septem* sp. nov., we treat these specimens as Labiobaetis cf. septem sp. nov., a MOTU (Molecular Operational Taxonomic Unit; see discussion in Kaltenbach et al. 2020a) with the same or nearly the same morphology than *Labiobaetis septem* sp. nov. Further investigations, including sequencing of nuclear genes, are necessary to clarify the status of this population.

### ﻿Biological aspects and the diversity of *Labiobaetis* in Thailand

Specimens were collected primarily from headwater and urban streams across a range of altitudes (10–810 m a.s.l.). Most streams were situated in forested areas with partially closed canopies, while a few were in urban environments with riparian vegetation and partially covered canopies. In headwater streams, the substrate was predominantly composed of gravel and sand. In contrast, urban streams had sandy substrates, with *Hydrilla verticillata* (L.f.) Royle (commonly known as Water Thyme) as dominant aquatic vegetation. The larvae were found in the flowing water.

The genus *Labiobaetis* was the most dominant and widely distributed baetid genus across all sampling sites in Thailand. It was present in every locality surveyed and consistently comprised multiple species at each site. In some cases, up to 100 specimens were collected per sampling, indicating that *Labiobaetis* is the most abundant genus within the family Baetidae in Thailand.

Considering the general mega-biodiversity in Thailand, and the obvious richness of *Labiobaetis* in this region, we have to expect many more new species with further studies in the future, despite the high collection efforts already done in the past years.

## Supplementary Material

XML Treatment for Labiobaetis multus

XML Treatment for Labiobaetis mon

XML Treatment for Labiobaetis lahu

XML Treatment for Labiobaetis tenasserimensis

XML Treatment for Labiobaetis angularis

XML Treatment for Labiobaetis tonsator

XML Treatment for Labiobaetis cf. paraoperosus

XML Treatment for Labiobaetis nisaratae

XML Treatment for Labiobaetis karen

XML Treatment for Labiobaetis septem

XML Treatment for Labiobaetis ranongensis

## ﻿Additional information

### Use of AI

No use of AI was reported.

### Author ORCIDs

Thomas Kaltenbach https://orcid.org/0000-0001-8052-0388

Jean-Luc Gattolliat https://orcid.org/0000-0001-5873-5083

Boonsatien Boonsoong https://orcid.org/0000-0002-8166-0021

Chanaporn Suttinun https://orcid.org/0000-0002-0380-1554

### Data availability

All of the data that support the findings of this study are available in the main text or Supplementary Information.

## Supplementary materials

Supplementary material 1 *Labiobaetis* species of Thailand, genetic distances (COI, K2P) Data type xls https://binary.pensoft.net/file/1459126This dataset is made available under the Open Database License (http://opendatacommons.org/licenses/odbl/1.0/). The Open Database License (ODbL) is a license agreement intended to allow users to freely share, modify, and use this Dataset while maintaining this same freedom for others, provided that the original source and author(s) are credited.Thomas Kaltenbach, Jean-Luc Gattolliat, Boonsatien Boonsoong, Chanaporn Suttinun

## References

[B1] Ball SL Hebert PDN Burian SK Webb JM (2005) Biological identifications of mayflies (Ephemeroptera) using DNA barcodes. Journal of the North American Benthological Society 24(3): 508–524. 10.1899/04-142.1

[B2] Barber-James HM Sartori M Gattolliat J-L Webb J (2013) World checklist of freshwater Ephemeroptera species. http://fada.biodiversity.be/group/show/35

[B3] Bespalaya YV Palatov DM Gofarov MY Kondakov AV Kropotin AV Sousa R Taskinen J Inkhavilay K Tanmuangpak K Tumpeesuwan S Vikhrev IV Bolotov IN (2023) Associations of mayfly larvae with *Corbicula* clams. Biological Journal of the Linnean Society. Linnean Society of London 138(2): 169–193. 10.1093/biolinnean/blac143

[B4] Chakrabarty P Warren M Page LM Baldwin CC (2013) GenSeq: An updated nomenclature and ranking for genetic sequences from type and non-type sources. ZooKeys 346: 29–41. 10.3897/zookeys.346.5753 PMC3821064 24223486

[B5] Folmer O Black M Hoeh W Lutz R Vrijenhoek R (1994) DNA primers for amplification of mitochondrial cytochrome c oxidase subunit I from diverse metazoan invertebrates. Molecular Marine Biology and Biotechnology 3: 294–299. http://www.mbari.org/staff/vrijen/PDFS/Folmer_94MMBB.pdf 7881515

[B6] Gattolliat J-L (2012) Two new genera of Baetidae (Ephemeroptera) from Borneo (East Kalimantan, Indonesia). Annales de Limnologie –International Journal of Limnology 48(2): 187–199. 10.1051/limn/2012012

[B7] Gattolliat J-L Cavallo E Vuataz L Sartori M (2015) DNA barcoding of Corsican mayflies (Ephemeroptera) with implications on biogeography, systematics and biodiversity. Arthropod Systematics & Phylogeny 73(1): 3–18. 10.3897/asp.73.e31813

[B8] Jacobus LM Macadam CR Sartori M (2019) Mayflies (Ephemeroptera) and their contributions to ecosystem services. Insects 10(6): 1–26. 10.3390/insects10060170 PMC6628430 31207933

[B9] Kaltenbach T Gattolliat J-L (2018) The incredible diversity of *Labiobaetis* Novikova & Kluge in New Guinea revealed by integrative taxonomy (Ephemeroptera, Baetidae). ZooKeys 804: 1–136. 10.3897/zookeys.804.28988 PMC6297208 30584389

[B10] Kaltenbach T Gattolliat J-L (2019a) A new species of *Tenuibaetis* Kang & Yang, 1994 from Indonesia (Ephemeroptera, Baetidae). ZooKeys 820: 13–23. 10.3897/zookeys.820.31487 PMC6361873 30728738

[B11] Kaltenbach T Gattolliat J-L (2019b) The tremendous diversity of *Labiobaetis* Novikova & Kluge in Indonesia (Ephemeroptera, Baetidae). ZooKeys 895: 1–117. 10.3897/zookeys.895.38576 31844411 PMC6906171

[B12] Kaltenbach T Gattolliat J-L (2020) *Labiobaetis* Novikova & Kluge in Borneo (Ephemeroptera, Baetidae). ZooKeys 914: 43–79. 10.3897/zookeys.914.47067 32132855 PMC7046705

[B13] Kaltenbach T Gattolliat J-L (2021) New species of *Labiobaetis* Novikova & Kluge from Southeast Asia and New Guinea (Ephemeroptera, Baetidae). ZooKeys 1067: 159–208. 10.3897/zookeys.1067.72251 34759723 PMC8575868

[B14] Kaltenbach T Garces JM Gattolliat J-L (2020a) The success story of *Labiobaetis* Novikova & Kluge in the Philippines (Ephemeroptera, Baetidae), with description of 18 new species. ZooKeys 1002: 1–114. 10.3897/zookeys.1002.58017 33363429 PMC7746671

[B15] Kaltenbach T Garces JM Gattolliat J-L (2020b) A new genus of Baetidae (Insecta, Ephemeroptera) from Southeast Asia. European Journal of Taxonomy 612(612): 1–32. 10.5852/ejt.2020.612

[B16] Kaltenbach T Garces J Gattolliat J-L (2021) *Philibaetis* gen. nov., a new genus from the Philippines (Ephemeroptera, Baetidae). Deutsche Entomologische Zeitschrift 68(1): 1–20. 10.3897/dez.68.59462

[B17] Kaltenbach T Garces J Gattolliat J-L (2022a) First contribution to *Labiobaetis* Novikova & Kluge in Cambodia (Ephemeroptera, Baetidae), with description of two new species. ZooKeys 1123: 63–81. 10.3897/zookeys.1123.90308 36762039 PMC9836613

[B18] Kaltenbach T Kluge NJ Gattolliat J-L (2022b) A widespread new genus of Baetidae (Baetidae, Ephemeroptera) from Southeast Asia. ZooKeys 1135: 1–59. 10.3897/zookeys.1135.93800 36761800 PMC9836713

[B19] Kaltenbach T Kluge NJ Gattolliat J-L (2023a) A new, widespread genus of Baetidae from South Asia (Insecta, Ephemeroptera). ZooKeys 1168: 231–266. 10.3897/zookeys.1168.104844 38318436 PMC10843360

[B20] Kaltenbach T Vuataz L Gattolliat J-L (2023b) New species of *Labiobaetis* Novikova & Kluge from New Guinea (Ephemeroptera, Baetidae): A never-ending story of diversity. Alpine Entomology 7: 83–134. 10.3897/alpento.7.106089

[B21] Kimura M (1980) A simple method for estimating evolutionary rates of base substitutions through comparative studies of nucleotide sequences. Journal of Molecular Evolution 16(2): 111–120. 10.1007/BF01731581 7463489

[B22] Kjærstad G Webb JM Ekrem T (2012) A review of the Ephemeroptera of Finnmark–DNA barcodes identify Holarctic relations. Norwegian Journal of Entomology 59(2): 182–195.

[B23] Kluge NJ (2004) The phylogenetic system of Ephemeroptera. Academic Publishers, Dordrecht, 1–442. 10.1007/978-94-007-0872-3

[B24] Kluge NJ (2005) Larval/pupal leg transformation and a new diagnosis for the taxon Metabola Burmeister, 1832 = Oligoneoptera Martynov, 1923. Russian Entomological Journal 13: 189–229.

[B25] Kluge NJ (2020a) Review of *Oculogaster* Kluge, 2016 (Ephemeroptera, Baetidae, *Procloeon* Bengtsson 1915). Zootaxa 4820(3): 401–437. 10.11646/zootaxa.4820.3.1 33056054

[B26] Kluge NJ (2020b) New subgenus Monilistylus subgen. n. and a new species Procloeon (Monilistylus) ornatipennis sp. n. (Ephemeroptera: Baetidae: *Procloeon*). Zootaxa 4742(3): 573–587. 10.11646/zootaxa.4742.3.11 32230372

[B27] Kluge NJ (2022) Two new species of *Waynokiops* Hill et al., 2010 (Ephemeroptera: Baetidae) from the Oriental Region. Zootaxa 5182(1): 41–63. 10.11646/zootaxa.5182.1.3 36095700

[B28] Kluge NJ Novikova EA (2014) Systematics of *Indobaetis* Müller-Liebenau & Morihara, 1982, and related implications for some other Baetidae genera (Ephemeroptera). Zootaxa 3835(2): 209–236. 10.11646/zootaxa.3835.2.3 25081445

[B29] Kluge NJ Novikova EA (2017) Occurrence of *Anafroptilum* Kluge 2012 (Ephemeroptera: Baetidae) in Oriental Region. Zootaxa 4282(3): 453–472. 10.11646/zootaxa.4282.3.2

[B30] Kluge NJ Suttinun C (2020) Review of the Oriental genus *Indocloeon* Müller-Liebenau 1982 (Ephemeroptera: Baetidae) with descriptions of two new species. Zootaxa 4779(4): 451–484. 10.11646/zootaxa.4779.4.1 33055765

[B31] Kluge NJ Godunko RJ Svitok M (2020) Nomenclatural changes in *Centroptella* Braasch & Soldán, 1980 (Ephemeroptera, Baetidae). ZooKeys 914: 81–125. 10.3897/zookeys.914.46652 32132856 PMC7046710

[B32] Kluge NJ Sivaruban T Srinivasan P Barathy S Isack R (2023) Diagnosis, variability, distribution and systematic position of *Labiobaetis pulchellus* (Müller-Liebenau & Hubbard,1985) (Ephemeroptera, Baetidae, *Baetis* s. l.). Zootaxa 5264: 94–108. 10.11646/zootaxa.5264.1.6 37044961

[B33] Lugo-Ortiz CR McCafferty WP Waltz RD (1999) Definition and reorganization of the genus *Pseudocloeon* (Ephemeroptera: Baetidae) with new species descriptions and combinations. Transactions of the American Entomological Society 125: 1–37.

[B34] McCafferty WP Waltz RD (1995) *Labiobaetis* (Ephemeroptera: Baetidae): new status, new North American species, and related new genus. Entomological News 106: 19–28.

[B35] Müller-Liebenau I (1984) New genera and species of the family Baetidae from West-Malaysia (River Gombak) (Insecta: Ephemeroptera). Spixiana 7: 253–284.

[B36] Pandiaran S Sivaruban T Sruthi SP Barathy S (2025) Rajasekaran Isack, Muthukumar Nadar MSA (2025) Two additional species of *Labiobaetis* Novikova & Kluge, 1987 (Ephemeroptera, Baetidae) from Tamil Nadu, India. Aquatic Insects 46(2): 81–93. 10.1080/01650424.2024.2443449

[B37] Phlai-ngam S Boonsoong B Gattolliat J-L Tungpairojwong N (2022a) *Megabranchiella* gen. nov., a new mayfly genus (Ephemeroptera: Baetidae) from Thailand with description of two new species. ZooKeys 1125: 1–31. 10.3897/zookeys.1125.90802 36761289 PMC9836710

[B38] Phlai-ngam S Tungpairojwong N Gattolliat J-L (2022b) A new species of *Alainites* (Ephemeroptera, Baetidae) from Thailand. Alpine Entomology 6: 133–146. 10.3897/alpento.6.96284

[B39] Phlai-ngam S Boonsoong B Gattolliat J-L Tungpairojwong N (2024) Taxonomic notes on the genus *Baetiella* Uéno, 1931 (Ephemeroptera, Baetidae), with description of three new species from Thailand. ZooKeys 1200: 303–352. 10.3897/zookeys.1200.116787 38766411 PMC11099474

[B40] Ridd M Barber A Crow M (2011) The Geology of Thailand. Geological Society, London. 10.1144/GOTH

[B41] Sanger F Nicklen S Coulson AR (1977) DNA sequencing with chain-terminating inhibitors. Proceedings of the National Academy of Sciences of the United States of America 74(12): 5463–5467. 10.1073/pnas.74.12.5463 271968 PMC431765

[B42] Sartori M Brittain JE (2015) Order Ephemeroptera. In: Thorp J Rogers DC (Eds) Ecology and general biology: Thorp and Corvich’s Freshwater Invertebrates.Academic Press, 873–891. 10.1016/B978-0-12-385026-3.00034-6

[B43] Shi W Tong X (2014) The genus *Labiobaetis* (Ephemeroptera: Baetidae) in China, with description of a new species. Zootaxa 3815(3): 397–408. 10.11646/zootaxa.3815.3.5 24943622

[B44] Shorthouse DP (2010) SimpleMappr, an online tool to produce publication-quality point maps. [Retrieved from https://www.simplemappr.net, Accessed March 03, 2021]

[B45] Sivaruban T Srinivasan P Barathy S Isack R (2022) A new species and record of Labiobaetis Novikova & Kluge, 1987 (Ephemeroptera: Baetidae) from India. Aquatic Insects 43(4): 319–334. 10.1080/01650424.2022.2070217

[B46] Sutthinun C Gattolliat J-L Boonsoong B (2018) A new species of *Platybaetis* Müller-Liebenau, 1980 (Ephemeroptera: Baetidae) from Thailand, with description of the imago of *Platybaetis bishopi* Müller-Liebenau, 1980. Zootaxa 4378(1): 85–97. 10.11646/zootaxa.4378.1.5 29690018

[B47] Suttinun C (2021) Systematics of family Baetidae (order Ephemeroptera) in southern and western Thailand. Doctoral thesis, Kasetsart University, Bangkok, 170 pp.

[B48] Suttinun C Gattolliat J-L Boonsong B (2020) *Cymbalcloeon* gen. nov., an incredible new mayfly genus (Ephemeroptera: Baetidae) from Thailand. PLoS One 15(10): 1–17. 10.1371/journal.pone.0240635 PMC7553360 33048998

[B49] Suttinun C Kaltenbach T Gattolliat J-L Boonsoong B (2021) A new species and first record of the genus *Procerobaetis* Kaltenbach & Gattolliat, 2020 (Ephemeroptera, Baetidae) from Thailand. ZooKeys 1023: 13–28. 10.3897/zookeys.1023.61081 33776512 PMC7969585

[B50] Suttinun C Gattolliat J-L Boonsoong B (2022) First report of the genus *Tenuibaetis* (Ephemeroptera, Baetidae) from Thailand revealing a complex of cryptic species. ZooKeys 1084: 165–182. 10.3897/zookeys.1084.78405 35233168 PMC8825428

[B51] Tamura K Stecher G Kumar S (2021) MEGA 11: Molecular evolutionary genetics analysis version 11. Molecular Biology and Evolution 38(7): 3022–3027. 10.1093/molbev/msab120 33892491 PMC8233496

[B52] Tofilski A (2018) DKey software for editing and browsing dichotomous keys. ZooKeys 735: 131–140. 10.3897/zookeys.735.21412 PMC5904324 29674865

[B53] Tungpairojwong N Phlai-ngam S Jacobus LM (2022) A new species of *Acentrella* Bengtsson, 1912 (Ephemeroptera: Baetidae) from Thailand. Zootaxa 5125(4): 351–378. 10.11646/zootaxa.5125.4.1 36101209

[B54] Vuataz L Sartori M Wagner A Monaghan MT (2011) Toward a DNA taxonomy of Alpine *Rhithrogena* (Ephemeroptera: Heptagenidae) using a mixed Yule-Coalescent Analysis of mitochondrial and nuclear DNA. PLoS One 6(5): 1–11. 10.1371/journal.pone.0019728 PMC3096624 21611178

